# Thermodynamics of Molecular Binding and Clustering in the Atmosphere Revealed through Conventional and ML-Enhanced Umbrella Sampling

**DOI:** 10.1021/acsomega.5c05634

**Published:** 2025-08-18

**Authors:** Jakub Kubečka, Yosef Knattrup, Georg Baadsgaard Trolle, Bernhard Reischl, August Smart Lykke-Møller, Jonas Elm, Ivo Neefjes

**Affiliations:** † 610542 Aarhus University, Department of Chemistry, Langelandsgade 140, Aarhus DK 8000, Denmark; ‡ 3835 University of Helsinki, Institute for Atmospheric and Earth System Research (INAR)/Physics P.O.Box 64, Helsinki FI 00014, Finland

## Abstract

Accurately modeling the binding free energies associated with molecular cluster formation is critical for understanding atmospheric new particle formation. Conventional quantum-chemistry methods, however, often struggle to describe thermodynamic contributions, particularly in systems exhibiting significant anharmonicity and configurational complexity. We employed umbrella sampling, an enhanced-sampling molecular dynamics technique, to compute Gibbs binding free energies for clusters formed from a diverse set of new particle formation precursors, including sulfuric acid, ammonia, dimethylamine, and water. By performing umbrella sampling along the evaporation coordinate, using forces computed at the semiempirical GFN1-xTB level of theory, we effectively capture entropic effects such as vibrational anharmonicities and transitions between different configurational minima, while avoiding errors from symmetry overcounting. In addition, we explored machine-learning-enhanced umbrella sampling simulations using neural network potentials trained on higher-level quantum chemistry data, demonstrating the feasibility of this approach for improving accuracy while maintaining computational efficiency. Our results show improved agreement with experimental values compared to conventional methods. We also present examples of gas-to-particle uptake processes, providing insights into cluster and aerosol–surface chemistry using first-principles approaches rather than commonly used molecular-mechanics force fields. This study demonstrates the importance of accounting for dynamics in predicting molecular binding thermodynamics in complex environments and highlights the potential of combining physics-based simulations with machine learning for reliable and scalable predictions.

## Graphical abstract

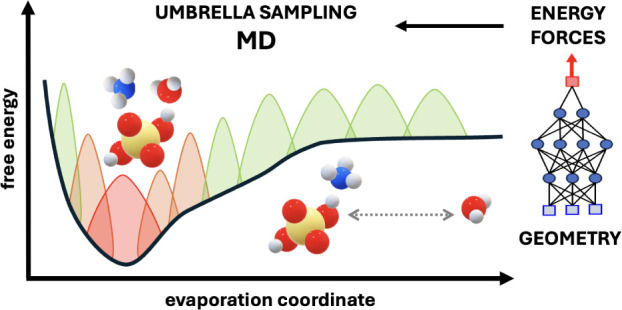

## 1 Introduction

Accurate characterization of intermolecular interactions and chemical reactions is of key importance across many scientific disciplines, including atmospheric sciences. Of particular interest is the binding and aggregation of gas-phase molecules into molecular clusters, which can subsequently grow through condensation and coagulation into aerosol particles.^1–5^ This process, known as new particle formation (NPF), plays an important role in shaping atmospheric composition and influencing climate.^6,7^

Despite extensive experimental^3,5,8–10^ and field studies,^8,11,12^ the earliest stages of NPF remain difficult to capture due to technical limitations. These include detection constraints of particle-counting instruments, which typically cannot detect clusters smaller than 2 nm in diameter, low concentrations of clusters and their precursor molecules, and the wide range of time scales associated with cluster binding and evaporation.^13,14^ For example, evaporation rates for atmospheric molecular clusters can span many orders of magnitude, from as low as 10^–20^ s^–1^ for strongly bound clusters to as high as 10^10^ s^–1^ for weakly bound clusters.^15–17^

Computational chemistry can offer valuable insights into these early stage processes. Quantum chemical (QC) methods have been employed to characterize potential energy surfaces and calculate properties, such as Gibbs binding free energies, for molecular clusters typically containing up to 10 molecules.^2,18–21^ The Gibbs binding free energies can be used to derive evaporation rates via the detailed balance equation.^22^ Recent advances have extended these methods to large clusters, reaching sizes of up to 2 nm in diameter, bridging the gap between theoretical modeling and experimental observations.^4,23–26^

In the current state-of-the-art, configurational sampling (CS) is used to identify the lowest free energy structure, for which the binding free energy is then calculated at a high level of theory. However, applying this approach to large molecular clusters presents several issues, including the high computational cost of accurate QC methods, the need for sophisticated CS strategies,^2,4,27–29^ and the inaccurate treatment of thermodynamic contributions. The latter issue stems from the neglect of: (1) local anharmonicity, caused by common statistical thermodynamics approaches relying on the harmonic oscillator (HO) approximation, which leads to significant errors in larger systems due to the increasing number of vibrational degrees of freedom; (2) global anharmonicity, resulting from considering only the lowest free energy conformer, thereby neglecting contributions from other significantly populated low-lying minima; (3) rotational symmetry, which can be incorrectly identified when quantum chemistry programs fail to detect molecular symmetry due to limited numerical precision and too low symmetry thresholds.

The impact of these errors depends on the binding strength and flexibility of the studied clusters. Besel et al.^30^ demonstrated that correcting for symmetry numbers is important, yet accurately estimating them for clusters can be challenging. Identifying the correct rotational symmetry number, particularly for monomers, is crucial for obtaining reliable absolute binding free energies. However, the rotational symmetry number becomes ambiguous in the presence of low-energy-barrier internal rotations (e.g., methyl group rotations), fluxional molecules that undergo rapid changes in symmetry, symmetry breaking due to anharmonicity (e.g., Jahn–Teller distortions), and the treatment of torsional modes as vibrations rather than free rotations, which is part of the low-vibrational frequency problem.

Halonen et al.^31^ recently demonstrated that for large clusters, local anharmonicity often outweighs global anharmonicity, as typically a single energy minimum is significantly populated. They also showed that local anharmonicity can influence binding free energies by up to *k*_B_ *T*/4 per vibrational degree of freedom at high temperatures. For example, Temelso et al.^32^ found that anharmonic corrections reduced the binding free energy of water clusters by 0.4–0.6 kcal/mol per water molecule. A common approach to address local anharmonicity in QC calculations is the use of vibrational scaling factors. However, for flexible systems or large molecular clusters (e.g., hydrogen-bonded networks) with strong anharmonicity, one scaling factor is not very reliable. Moreover, vibrational scaling factors are typically derived from small molecules where anharmonic effects may differ. While such empirical corrections can improve agreement with experimental data, truly predictive accuracy requires more sophisticated theoretical methods. Another limitation of the HO approximation is its inability to describe near-flat potentials, which results in artificially low frequencies. These are typically corrected using the quasi-harmonic approximation,^33^ which is likely another source of errors for these systems.^34^

Molecular dynamics (MD) enables the exploration of cluster dynamics, including transitions between configurations and anharmonic vibrational motions. However, standard MD simulations often become trapped in local minima. To overcome this limitation, enhanced sampling techniques such as umbrella sampling (US)^35,36^ can be employed to more thoroughly explore the relevant configurational space. When applied along a cluster’s evaporation coordinate, US provides the free energy profile along this pathway, from which the binding free energy can be computed.

In this study, we investigated the suitability of US for obtaining accurate binding free energies of atmospherically relevant cluster formation, including the entropic effects (e.g., local/global anharmonicity and symmetry) that are often inaccurately assessed in QC-based statistical thermodynamics. Instead of estimating free energies from harmonic approximations at a single optimized structure, US samples the full ensemble of thermally accessible structures, including multiple energy minima and the vibrational motion around them, without relying on harmonic assumptions. Rotational symmetry is implicitly accounted for, as symmetrically equivalent configurations are sampled with equal probability. We performed US simulations along the evaporation coordinate of a molecule from a cluster, defined as the distance between the center of mass of the evaporating molecule and that of the remaining cluster. Due to the high computational cost of QC methods, we employed the semiempirical GFN1-xTB method^37^ for these simulations. While GFN1-xTB does not yield quantitatively accurate binding free energies, it is expected to sufficiently capture the relative magnitude of entropic effects.

While the direct application of high-level QC methods in US is computationally prohibitive, recent advances in machine learning (ML) show promise in overcoming this computational limitation.^38–45^ ML potentials enable fast MD simulations while accurately reproducing the trained QC method. Bertazzo et al.^46^ incorporated ML into their US workflow, not to enhance MD simulations, but to facilitate post-MD analysis of minimum free energy path searches. Zhao et al.^47^ employed US with ML potentials trained at the DFT level to study solid-state phase transitions, and Bonati et al.^48^ demonstrated the use of neural networks (NNs) in other variational sampling approaches. Nevertheless, to the best of our knowledge, ML-enhanced US simulations have been unexplored beyond these studies. In this work, we, therefore, investigated the use of ML-enhanced US to improve the accuracy of binding free energy predictions.

In summary, we argue that the conventional statistical thermodynamics built on top of QC calculations fails to capture key entropic effects in molecular clusters. As an alternative, we propose a simulation strategy based on umbrella sampling: (A) to recover the missing entropic effects using a low level of theory, or (B) compute binding free energies from the simulation at high level of theory, here substituted by a more efficient machine learning potential. By investigating US as an alternative approach for calculating cluster binding free energies including entropic effects, as well as integrating ML techniques, this study lays the groundwork for future research on molecular cluster formation and stability, with potential applications beyond atmospheric chemistry into reaction dynamics and computational thermodynamics.

## 2 Theory

### 2.1 Gibbs Binding Free Energies from a Potential of Mean Force

For a system with a constant number of atoms *N*, at constant volume *V*, and temperature *T* (canonical ensemble), the Helmholtz free energy is given by

1 $$F\left(N,\;V,\;T\right)=U-TS=-k_{B}T\ln Q$$

where *U* is the internal energy, *S* the entropy, *k*_B_ the Boltzmann constant, and *Q* the canonical partition function. For a classical system of identical particles, the partition function can be calculated from an integral over phase space:

2 $$Q=\int \exp\left(-\beta H\left(q^{N},\;p^{N}\right)\right)dq^{N}dp^{N}=\frac{1}{h^{3N}N!}\int \exp\left(-\beta U\left(q^{N}\right)\right)dq^{N}$$

where {**q** ^*N*^, **p** ^*N*^} denotes the 6*N*-dimensional phase space of atomic coordinates and momenta, β = 1/(*k*_B_ *T*), $H\left(q^{N},p^{N}\right)=U\left(q^{N}\right)+\underset{i}{\sum }p_{i}^{2}/2m$ is the system’s Hamiltonian, and *h* is Planck’s constant. In the second equation the integration over the momenta has been carried out to leave an expression in terms of an integral over configuration space.

We consider a gas-phase system at standard thermodynamics conditions (*V* corresponding to *p* = 1 atm and *T* = 298.15 K), containing species *A* and *B* that undergo dimerization:

3 A+B⇌AB

The standard Helmholtz binding free energy Δ*F* ^⊖^ is the difference between the energies of the bound state *AB* and the unbound states *A* and *B*,

4 ΔF⊖=FAB⊖−FA⊖−FB⊖=−kBTln(QABQAQB)=−kBTln⁡Kbind,NVT⊖

where $K_{\mathrm{bind},NVT}^{⊖}$ is the canonical equilibrium constant, or association constant.

Direct evaluation of the partition functions in eq 4 becomes computationally impossible for complex interacting systems. Instead, a useful approach consists in calculating the Helmholtz free energy profile *F*(ξ) as a function of a collective variable (CV) or reaction coordinate ξ, which allows to distinguish the different states of interest^49^ (e.g., bound and unbound), while ξ depends on the Cartesian coordinates as ξ­(**q** ^*N*^):

5 F(ξ)=−kBTln⁡∫δ[ξ−ξ(qN)]·exp(−βU(qN))dqN=−kBTln⁡∫exp(−βw(ξ))·J(ξ)dξ

where δ is the delta distribution, *w*(ξ) is the potential of mean force (PMF), and the Jacobian determinant *J*(ξ) accounts for how the volume in configuration space changes under the transformation ξ­(**q** ^*N*^), effectively adjusting the measure d**q** ^*N*^ to dξ. The PMF is the thermally averaged potential energy along ξ, which can be sampled from the corresponding statistical ensemble using atomistic MD or Monte Carlo simulations. To ensure proper sampling in the presence of free energy barriers or large changes in free energy along ξ, biased sampling techniques such as umbrella sampling (US),^35^ presented in Section 2.3, are typically employed.

For the calculation of binding free energies between molecules or clusters, a natural choice for the CV, ξ, is the center of mass distance *r* between *A* and *B*. If the PMF *w*(*r*) is known, then $K_{\mathrm{bind},NVT}^{⊖}$ can be calculated as^50–53^

6 $$K_{\mathrm{bind},NVT}^{⊖}=\frac{4\pi }{V^{⊖}}\int_{\mathrm{bound}}r^{2}\exp\left(-\beta w\left(r\right)\right)dr$$

where *V* ^⊖^ ≈ 41 nm^3^ is the molecular volume at standard thermodynamics conditions and the factor 4π*r* ^2^ corresponds to the Jacobian determinant from the transformation between the radial CV *r* and Cartesian coordinates. As noted by Duboué-Dijon and Hénin,^51^ the integration in eq 7 depends on the definition of the bound state. For relatively strongly bound systems, eq 7 is, however, largely insensitive to the exact bound state definition (see Section S1). In this work, the upper bound of the definite integral was taken as the value *r*_max_, where the free energy profile *w*(*r*) – *k*_B_ *T* ln­(4*π r* ^2^) reaches a maximum, which arises from the competition of the PMF at short distances and the configurational entropy gain at large distances. As the PMF quickly becomes very repulsive at short distances, the integrand is essentially zero, and we can therefore extend the lower bound of the integration to *r* = 0. The Helmholtz binding free energy can then be calculated from the PMF as

7 $$\Delta F^{⊖}=-k_{B}T\;\ln\frac{4\pi }{V^{⊖}}\int_{0}^{r_{\max}}r^{2}\;\exp\left(-\beta w\left(r\right)\right)dr$$

In gas-phase chemistry, the isobaric–isothermal ensemble, where *p* is constant instead of *V*, is the more appropriate choice, and here we consider Gibbs free energies,

8 G(N,p,T)=F(N,V,T)+pV

The Gibbs binding free energy is obtained from the Helmholtz binding free energy as

9 $$\Delta G^{⊖}=G_{AB}^{⊖}-G_{A}^{⊖}-G_{B}^{⊖}=\Delta F^{⊖}+p\Delta V=\Delta F^{⊖}-k_{B}T$$

where we have used the ideal gas law *p*Δ*V* = Δ*Nk*_B_ *T* with Δ*N* = −1 for dimerization. The binding free energy should be further corrected for symmetry. For instance, in the case of homodimerization (*A* + *A* ⇌ *A*_2_):^51^

10 $$\Delta G_{\mathrm{homo}}^{⊖}=\Delta G^{⊖}+k_{B}T\;\ln2$$

The same correction applies to *A* + *B*_1_ *A*_1_ ⇌ *B*_1_ *A*_2_ and the factor increases with system size (e.g., *k*_B_ *T* ln 3 for formation of B_*x*_A_3_). This can be understood as a correction for all possible *A* molecule evaporations from the cluster. All reported free energies refer to standard conditions, but for brevity, we omit the superscript ⊖ throughout the remainder of the article.

#### 2.1.1 Quantum Correction

Particles in a quantum potential well occupy discrete vibrational levels.^54^ Figure 1 shows these levels for water dimerization obtained by solving the 1-D Schrödinger equation for its PMF.

**Figure 1:**
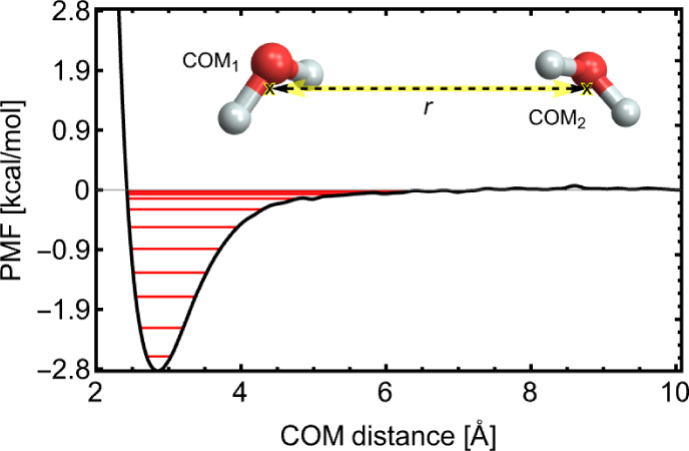
Potential of mean force (PMF) for water dimerization along the center of mass (COM) distance. The vibrational energy levels obtained from solving the Schrödinger equation for the 1-dimensional anharmonic PMF are indicated with red lines. Further details on the PMF calculations are presented in the Results section.

The classical integration of the PMF is inaccurate in the potential well, where the colliding species are close, and should be corrected to account for quantum effects. While this correction is included in our study, detailed analysis in Section S2 shows that its overall impact on free energies at room temperature is negligible (less than 0.03 kcal/mol). However, we note that this correction becomes significant at low temperatures (*T* < 200 K), which is less relevant for studies at atmospheric conditions.

### 2.2 Gibbs Free Energies from Quantum Chemistry

Unlike molecular dynamics simulations, where Δ*G* is determined by tracking the system’s dynamical behavior, QC calculations typically involve identifying a stationary potential energy minimum through CS and geometry optimization, followed by solving the electronic Schrödinger equation for this equilibrium structure. The free energy at a specific temperature is then obtained in an ad hoc manner by applying thermal and entropic corrections to the electronic energy, under the assumption that the free energy minimum coincides with the electronic energy minimum. These corrections account for translational, rotational, and vibrational contributions, and are calculated by assuming the system resides solely in its electronic ground state, follows the ideal gas law, undergoes rigid rotor rotations, and exhibits harmonic oscillator vibrations.^55^

In this work, we used a well-established CS approach, routinely employed by our group.^2,27^ Searching for the global free energy minimum is essential, as this structure is assumed to be the most populated and, therefore, contributes the most to the system’s total free energy. Several studies,^27,30,56^ have shown that Boltzmann averaging over the energy levels of other low-lying minima typically lowers the absolute Gibbs free energy by no more than 0.3 kcal/mol. As a result, many studies focus solely on accurately characterizing the global free energy minimum, an approach we also adopt in this work. Subsequently, each molecular system treated via the QC framework is geometrically optimized using the ωB97X-D/6–31++G­(d,p)^57^ method in Gaussian 16 B.01,^58^ hereafter referred to as DFT.

Vibrational frequency calculations and ad hoc thermal corrections are calculated at the same level of theory. Low vibrational frequencies are treated using the Grimme quasi-rigid-rotor harmonic oscillator approach,^33^ with a frequency threshold of 100 cm^–1^. The electronic energy is further refined through a single-point energy calculation at DLPNO–CCSD­(T_0_)/aug-cc-pVTZ^59–61^ with NormalPNO criteria in ORCA 5.0.4.^62–64^ In this work, properties obtained through this workflow are denoted as DLPNO//DFT.

Our workflow follows a widely used high-level QC approach that often serves as the final level of theory for molecular clusters in the atmospheric computational chemistry community.^2,20,65–72^ In the Results section, QC calculations at the lower GFN1-xTB^37^ level of theory are calculated with the xtb program^73^ and are denoted as XTB.

### 2.3 Umbrella Sampling

Umbrella Sampling (US)^35,74^ is a well-established biased sampling technique^49^ used to compute free energy changes as a function of a CV. In this work, we employed US using the cluster evaporation coordinate *r* as the CV, defined as the distance between the center of mass of the evaporating molecule and that of the remaining cluster. For dimers, this reduces to the distance between the centers of mass of the two molecules. We performed multiple independent simulations, each incorporating a harmonic umbrella potential added to the system’s Hamiltonian, centered at points *r*_*i*_ along the CV:

11 $$V_{i}=\frac{k_{\mathrm{bias}}}{2}{\left(r_{i}-r\right)}^{2}$$

where *k*_bias_ denotes the force constant and *r*_*i*_ is the position along the CV in the *i*-th umbrella window. This approach ensures proper sampling of the biased distribution $P_{i}^{b}$ ­(*r*) within a specific window.

If there is sufficient overlap between the biased distributions $P_{i}^{b}$ –(*r*) obtained in neighboring windows, the unbiased distribution *P* ^u^(*r*) and associated free energy profile *F*(*r*) within a certain range of the CV *r* can be reconstructed through self-consistent histogram reweighting techniques, such as WHAM^75^ and umbrella integration (UI).^76^ In this work, we used the latter method, as implemented in the umbrella_integration code.^76–78^ UI reconstructs the free energy profile by numerically integrating the mean force obtained from each window.^54,79^ The PMF *w*(*r*) is obtained by subtracting the entropic contribution *k*_B_ *T*ln­(4π*r* ^2^) from the obtained free energy profile. This entropic term arises from mapping the free energy onto a radial collective variable and accounts for the increase in the number of available microstates with increasing distance *r*.

All US simulations were performed using the Jammy Key (JK) framework,^80^ which integrates the Atomic Simulation Environment (ASE^81^ with computationally efficient XTB^37^ calculators such as tblite^82^ and xtb-python,^83^ as well as a ML calculator trained on various levels of theory. Employing a quantum Hamiltonian enables the simulation of chemical reactions, including atmospherically relevant proton transfers.

Our US workflow consists of the following steps:

(1) **Structural screening**: Unbiased MD simulations are performed to generate a pool of random configurations.
(2) **Equilibration**: In separate simulations, the random configurations are separated according to the US windows and equilibrated under the influence of a thermostat and the corresponding US bias.
(3) **Production**: The actual US simulations

Ten independent production runs are performed, and the biased distributions, $P_{i}^{b}$ ­(*r*), are combined for each window to improve configurational space sampling. Finally, the unbiased PMF is obtained using UI, which is then integrated (see Section 2.1) to compute Δ*G*. The individual steps and their simulation parameters are summarized in Table 1. A detailed technical description of each parameter, along with its validation analysis, is provided in Section S3.

**Table 1:** Summary of All MD Simulations Used in This Work

	Screening	Equilibration	Production Run
time step Δ*t*	1 fs	1 fs	1 fs
steps *n*	100k = 10^5^	100k = 10^5^	500k = 5 × 10^5^
thermostat^84,85^	Langevin	Langevin	CSVR/Langevin
coupling	0.01 fs^–1^	0.01 fs^–1^	0.04/0.01 fs^–1^
target *T*	300 K	300 K	300 K
constraints	(ext. force)^optional^	(ext. force)^optional^	(ext. force)^optional^
US constraints	—	US harmonic bias	US harmonic bias
*k*_bias_	—	$\frac{\min\left(n,1000\right)}{1000}\cdot 100\;\mathrm{kcal/mol/Å}$	100 kcal/mol/Å
force	—	–*k*_bias_(1 – ξ_bias_/Δ)·Δ⃗	–*k*_bias_(1 – ξ_bias_/Δ)·Δ⃗
initial velocities	Maxwell–Boltzmann	Maxwell–Boltzmann	—
initial position	[0,0,0]	Δ = max(ξ_bias_,1.5 ∑ *R*_*i*_)	—
calculator	GFN1-xTB	GFN1-xTB or PaiNN	GFN1-xTB or PaiNN
repetitions	1	10 for each window	10 for each window

### 2.4 Machine-Learning Enhancement

In previous work,^26^ we showed the applicability of the polarizable atom interaction NN (PaiNN,^86^ as implemented in SchNetPack 2.0 ^87,88^ to reproduce the energies and forces of clusters containing sulfuric acid (SA) and ammonia (AM) with up to 30 molecules. For SA_15_AM_15_ clusters, the learning errors were as low as 0.6 kcal/mol for energies and 0.2 kcal/mol/Å for forces. In this work, we employ the same NN architecture with similar hyperparameters (see Section S4). A single NN was trained per system. Improved accuracy might be obtained by ensemble averaging across multiple networks,^26,41^ but this will be explored in future work. Here, we focus instead on exploring different approaches for constructing the training data set.

Training data for the NN were prepared separately for each system. Figure 2 illustrates the construction of the training database, which begins with short US simulations at the XTB level, initialized from randomly sampled monomer geometries. These simulations were performed at both 300 and 500 K to better capture high-energy structures. Approximately 80 unique structures^27^ were sampled from each US window and across multiple repeat simulations. This approach likely samples a configurational space similar to the one explored in subsequent MD simulations at the target QC level. To further refine the description of individual molecules, we employed the Adaptive Density-Guided Approach (ADGA),^89^ implemented in MidasCpp,^90^ to sample the potential energy surface of monomers along their normal modes (see Section S5 for details). For all configurations, the energy and its gradient were evaluated at the desired QC level. Subsequently, the NN was trained on this data set and used for the US simulations.

**Figure 2:**
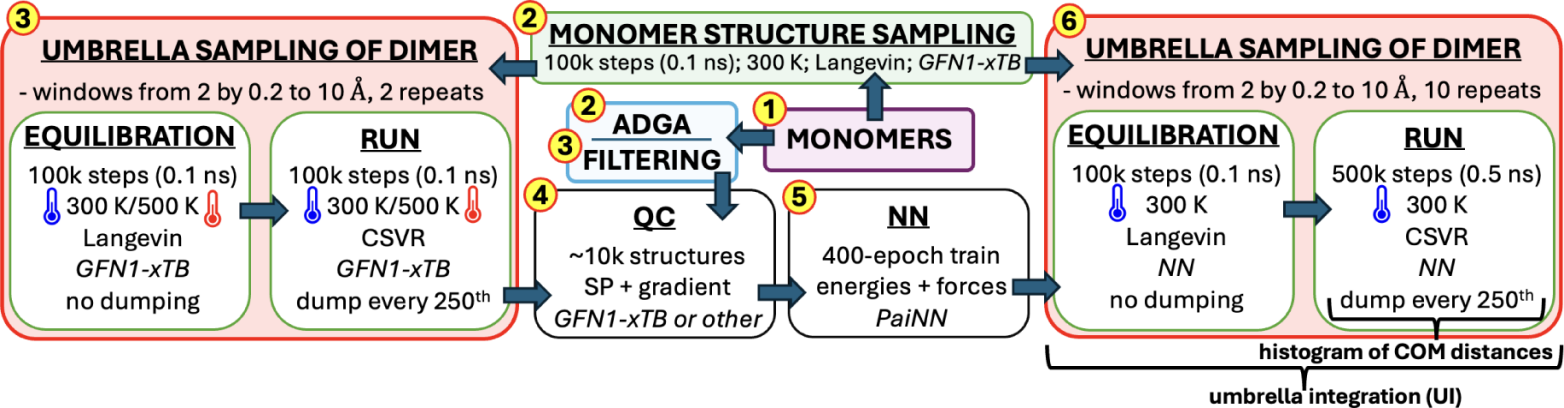
Scheme for fully automated generation of quantum chemistry (QC) data for machine learning (NN = neural network) followed by ML-enhanced US with the accuracy of a desired QC method.

This offers a fully automated approach for computing the PMF at a high QC level while maintaining a reasonable computational time. We note that for high levels of theory, the slowest step in this procedure is the QC step, which is, on the other hand, well-parallelizable. Moreover, when studying multiple similar reactions (e.g., fragmentation of various sulfuric acid–ammonia cluster sizes), a single NN can be used for all systems.

## 3 Results and Discussion

### 3.1 Umbrella Sampling Methodology Tests

To assess the accuracy and limitations of our umbrella sampling (US) approach, we performed the following tests:

- **Noninteracting particles**: A US simulation of two noninteracting particles results in a flat potential of mean force (PMF) with numerical errors <0.1 kcal/mol, as expected. At distances below ∼0.5 Å, larger deviations (up to 0.3 kcal/mol) appear because the distance is strictly positive, causing the bias potentials to become asymmetric near zero. Further discussion is provided in Section S6.
- **Two interacting atoms**: A US simulation of two atoms yields a PMF that corresponds to their dissociation curve at the XTB level. Moreover, for argon–argon collision, the free energy obtained from PMF integration exactly matches the QC^XTB^ value, demonstrating that, in the absence of entropic contributions, US reproduces the QC result. Further details are provided in Section S7.
- **Molecular binding with single dimer configuration**: Formic acid dimerization results in a single significantly populated dimer configuration. The Gibbs binding free energy from US closely matches the QC^XTB^ value, indicating that local anharmonicity plays a minor role in this case. These results are further detailed in Section 3.2.
- **Binding of other molecules**: For the binding of two molecules, such as sulfuric acid + water, sulfuric acid + ammonia, and even water dimerization, the Gibbs binding free energy from US differs significantly from the QC^XTB^ value. We attribute this discrepancy mainly to neglected entropic effects. This is further discussed in Section 3.2.
- **Thermostat**: Thermostats adjust the system’s kinetic energy to maintain a target temperature. This inherently alters the dynamics of the system. Halonen et al.^31^ noted that the canonical sampling through velocity rescaling (CSVR) thermostat better preserves realistic molecular trajectories compared to, e.g., the stochastic Langevin thermostat. However, in US, the focus is not on reproducing accurate trajectories but on sampling a representative configuration distribution under the applied bias. We compared CSVR and Langevin thermostats during the production run of several systems and found negligible differences in Δ*G* (<0.15 kcal/mol). Further details are provided in Section S8.
- **Unwanted evaporations**: In US simulations involving a weakly bound cluster, the cluster evaporation can occur during the simulation. To prevent this, an external potential can be applied. In this work, we focus on strongly bound clusters, where such a potential is not required. However, studying systems like (methanol)_3_ would necessitate an external potential. Addressing this goes beyond the scope of the present study, though we briefly comment on it in Section S9. An external potential is only employed in this work to construct the free energy profile in Section 3.3.
- **US simulation length**: To ensure well-converged Δ*G* values and smooth PMFs, we combine 10 independent simulations of 0.5 ns each, resulting a total simulation time equivalent of 5 ns per US production run. Extending the simulation time beyond this did not significantly reduce the variance in Δ*G*. We found that a 2 ns simulation time equivalent yields errors <0.05 kcal/mol for water dimerization and ± ∼0.7 kcal/mol for the (sulfuric acid)_1_+(sulfuric acid)_3_(ammonia)_4_ addition. See more details in Section S10.

### 3.2 Clustering Free Energies

In the following sections, we examine binding/clustering involving the following molecules: W = water, MeOH = methanol, DMA = dimethylamine, TMA = trimethylamine, EDA = ethylenediamine, GD = guanidine, ACA = acetic acid, FA = formic acid, SA/B^–^ = sulfuric acid/bisulfate, and NTA/NT^–^ = nitric acid/nitrate.

#### 3.2.1 Comparison to Experiments

Experimental measurements of cluster binding free energies are challenging. Nonetheless, we found several experimental studies reporting binding free energies or equilibrium constants for atmospherically relevant systems (see Section S11 for numerical values).^72,91–111^ Following the steps described in Section 2.3, we performed US simulations for these systems. The resulting PMFs are shown in Section S12, and a representative subset is highlighted in Figure 3.

**Figure 3:**
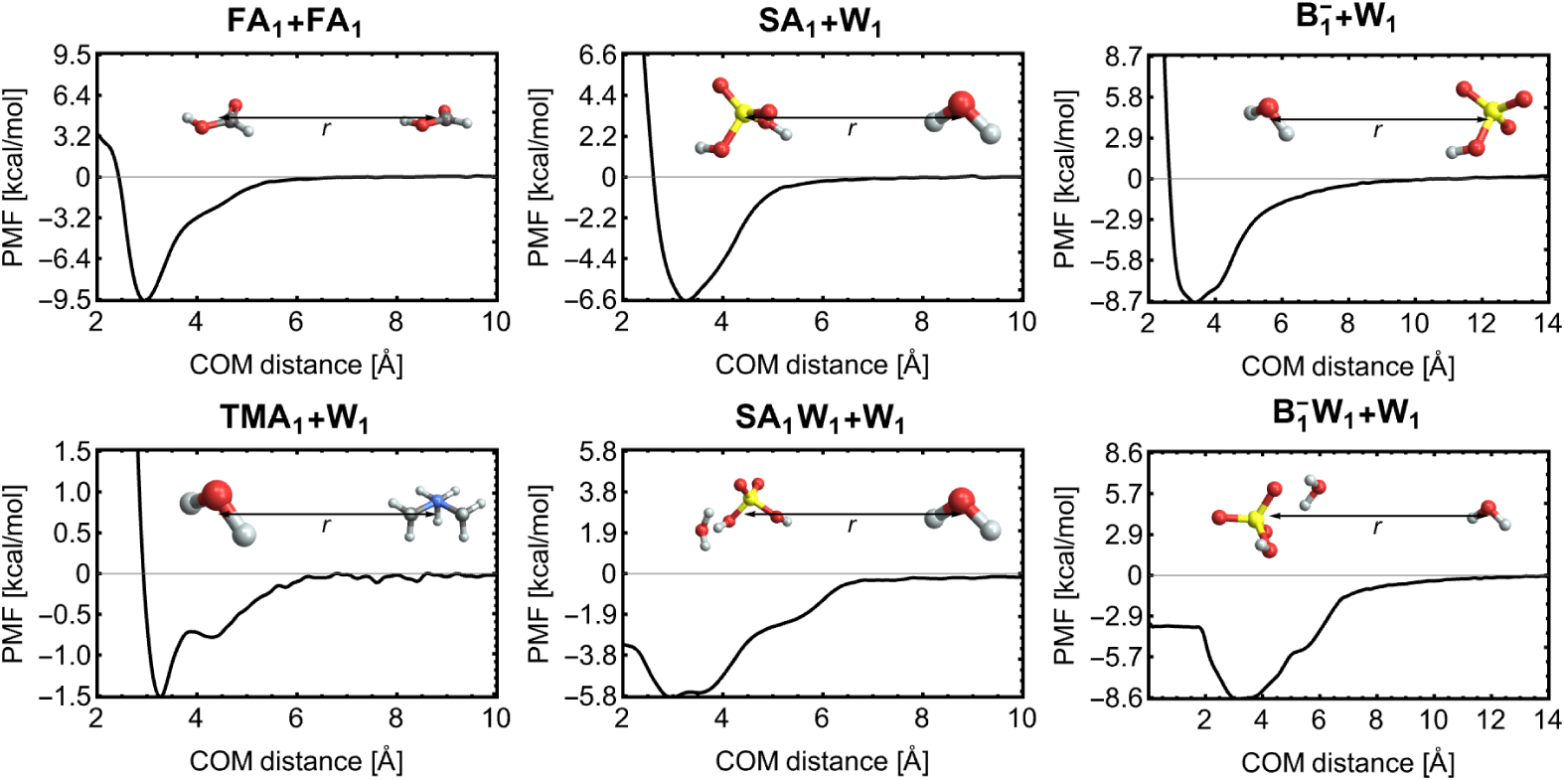
Potentials of mean force (PMF) obtained via US simulations. The evaporation coordinate corresponds to the center-of-mass (COM) distance. Legend: W = water, TMA = trimethylamine, FA = formic acid, and SA/B^–^ = sulfuric acid/bisulfate.

Each PMF exhibits a pronounced free energy well. Systems like TMA_1_+W_1_ and SA_1_W_1_+W_1_ display multiple minima within the well, corresponding to different binding configurations. Some systems have a single distinct minimum, while others (e.g., flexible systems) have multiple minima merged into a single broad minimum. Through the integration in eq 4, the well shape and depth are important in determining Δ*G*.

Since the well depth is given as the difference between the PMF value at the minimum and at large distances, we fitted the asymptotic tail of the PMF to either a Morse potential or a constant function to reduce errors from possible noise in the tail (see, e.g., TMA_1_W_1_) and obtain a clear estimate of the well depth. We shift the PMF so that it asymptotically approaches zero as the colliding species move away from each other. It is important to note that for molecule–molecule collisions, the PMF goes to infinity as *r* → 0, due to the repulsion from forcing the molecules to occupy the same space. However, in cluster–molecule collisions (e.g., SA_1_W_1_+W_1_ and B_1_W_1_+W_1_), the incoming molecule can enter the cluster and reach *r* = 0. This is often accompanied by an increase in free energy, as placing the molecule at the cluster center generally does not correspond to the lowest free energy configuration.

Figure 4 shows the binding free energies Δ*G* obtained from the US simulations, QC methods, and experiments. Although the experimental values may be subject to considerable uncertainties, we assume that the experimental mean provides the correct value and evaluate the quality of each computational method based on the mean absolute error (MAE) relative to these values. The DLPNO//DFT statistical thermodynamics approach is known to systematically underbind clusters,^112^ resulting in overestimated binding free energies, with MAE = 1.38 kcal/mol. XTB performs worse with MAE = 1.66 kcal/mol and would likely yield even larger errors for systems with strong binding and large enthalpic contributions. Similarly, QC^XTB^ consistently overestimates Δ*G* compared to experiments, except in the case of FA and ACA dimerization. In contrast, US agrees better with experiments than both QC methods, with MAE = 0.71 kcal/mol. This indicates that accounting for thermodynamic effects neglected in standard QC calculations can yield lower errors relative to experiments. However, as cluster binding becomes stronger, the accuracy of the electronic binding energy becomes more critical than the entropic contributions, and XTB lacks the accuracy needed to capture this reliably. To address this, we combined the entropic correction estimated at the XTB level (US^XTB^ – QC^XTB^) with high-level QC^DLPNO//DFT^ electronic energies. This hybrid approach reaches an MAE of 0.77 kcal/mol. Performing US simulations at a high level of theory is computationally unfeasible. Hence, to enable US at an accurate QC level, we explore machine learning-enhanced US in Section 3.4.

**Figure 4:**
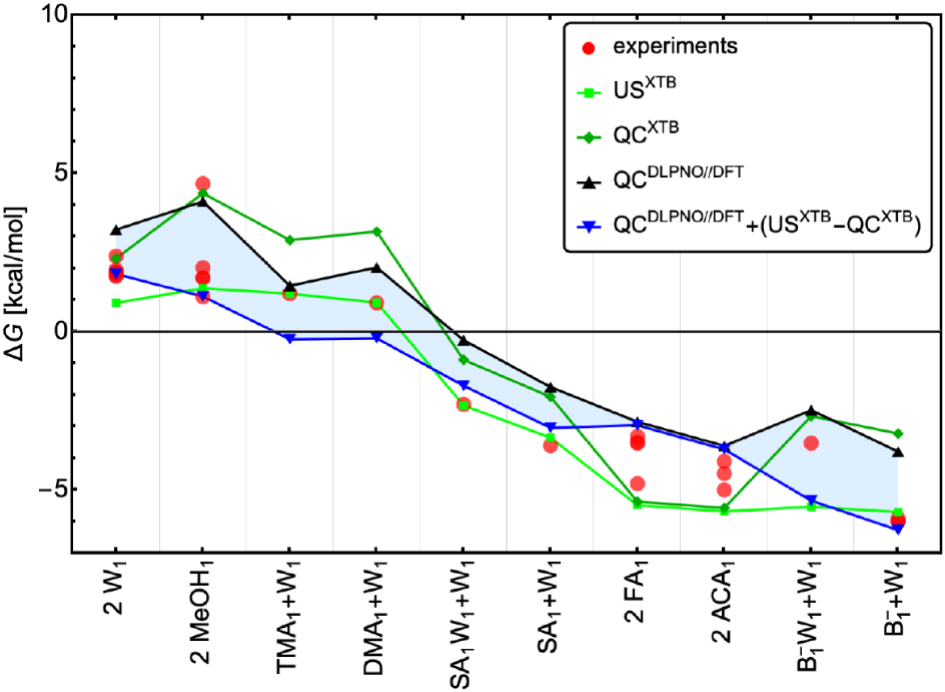
Gibbs binding free energies obtained from US simulations and traditional QC calculations at a low level of theory (XTB = GFN1-xTB) and at high level of theory (DLPNO//DFT = ωB97X-D/6–31++G­(d,p) with DLPNO^NormalPNO^–CCSD­(T_0_)/aug-cc-pVTZ single-point electronic energy correction). The references for the experimental values are mentioned in the main text. Legend: W = water, MeOH = methanol, DMA = dimethylamine, TMA = trimethylamine, ACA = acetic acid, FA = formic acid, and SA/B^–^ = sulfuric acid/bisulfate.

#### 3.2.2 Other Studied Systems

In this section, we examine additional systems relevant to new particle formation for which, to the best of our knowledge, no experimental binding free energy values have been published. Figure 5 presents the binding free energies obtained from QC and US, with the corresponding PMFs shown in Section S12. For systems such as SA_1_+SA_1_, SA_1_+AM_1_, SA_1_+DMA_1_, and SA_1_+SA_3_W_4_, the difference between QC^DLPNO//DFT^ with and without the entropic correction obtained at the XTB level is relatively small (<1.5 kcal/mol). However, even these modest differences can translate into approximately an order of magnitude variation in the evaporation rates. In contrast, for systems such as SA_1_+SA_3_AM_4_, SA_1_+SA_3_EDA_4_, $B_{1}^{-}$ +SA_1_, and N $T_{1}^{-}$ +NTA_1_, the binding free energies from US^XTB^ and QC^XTB^ deviate significantly, suggesting an increased importance of entropic contributions. As shown, the net entropic contribution can be either positive or negative, depending on the relative structural flexibility (i.e., configurational freedom) of the reactants and products. For instance, a positive entropic correction to the reaction free energy can arise when the interaction between functional groups of two reactants restricts internal rotations around involved bonds in the resulting cluster, effectively reducing its thermally accessible phase space. Such “locking” of degrees of freedom makes the product more rigid than the isolated reactants. However, we speculate that as cluster size increases, these individual structural effects tend to average out, and the entropic contribution becomes more systematic and predictable. The largest difference, 17.6 kcal/mol, is observed for SA_1_+SA_3_GD_4_, which appears suspicious. To verify, we repeated the US procedure for several systems using the GFN2-xTB level of theory (see Section S11). The obtained entropic contributions were similar to those obtained at GFN1-xTB, except for SA_1_+SA_3_GD_4_, where a correction of – 5.74 kcal/mol was found. We speculate that GFN1-xTB is not well parametrized for SA–GD interactions, leading to an inadequate potential energy surface that cannot provide reliable entropic corrections. For the other systems, the low level of theory appears to provide reasonable estimates of entropic contributions. Nonetheless, high-level US simulations could improve the reliability of the entropic contribution estimates, which will be addressed in Section 3.4.

**Figure 5:**
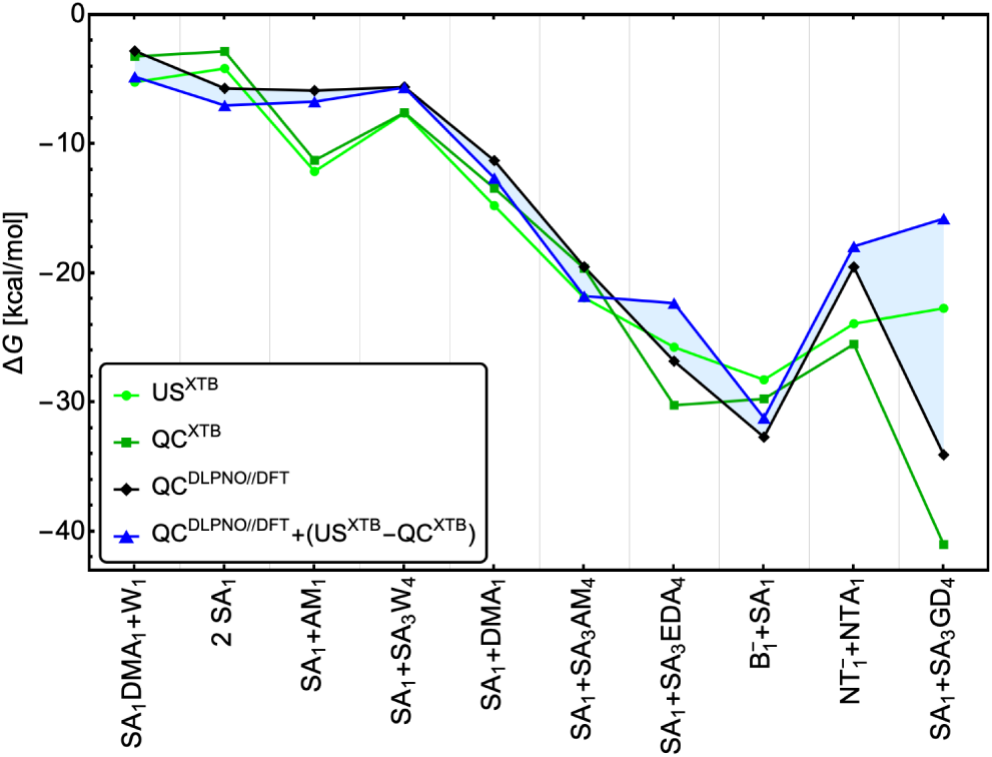
Gibbs binding free energies obtained from US simulations and traditional QC calculations at a low level of theory (XTB = GFN1-xTB) and at high level of theory (DLPNO//DFT = ωB97X-D/6–31++G­(d,p) with DLPNO^NormalPNO^–CCSD­(T_0_)/aug-cc-pVTZ single-point electronic energy correction). We have not found experimental values for these reactions. Legend: W = water, DMA = dimethylamine, EDA = ethylenediamine, GD = guanidine, SA/B^–^ = sulfuric acid/bisulfate, and NTA/NT^–^ = nitric acid/nitrate.

### 3.3 Gas-to-Particle Uptake

Aerosols play a crucial role in atmospheric processes, influencing climate, air quality, and chemical reactivity. PMF calculations based on US offer an effective approach to explore the free energy landscape associated with a molecule approaching an aerosol. Alongside other enhanced sampling methods—such as metadynamics,^113^ adaptive biasing force,^114^ steered MD simulations,^115^ and transition path sampling^116^—US can thus provide insights into key processes such as adsorption, dissolution, surface penetration, and reactive collisions.

Rather than modeling a full aerosol particle, we examined uptake onto a 10-molecule water cluster. However, the methodology is fully transferable to other clusters/aerosols. Water clusters are unstable and lead to undesired evaporation during simulations. Therefore, we applied a cluster-centered flat-bottom harmonic external potential (with *k*_EF_ = 1 kcal/mol/Å^2^ and varying range) to prevent “aerosol” fragmentation/evaporation (see Section S9).

Figure 6a presents the PMF for a water molecule colliding with the constrained cluster, calculated at the GFN-FF level of theory. GFN-FF was chosen for computational efficiency, though GFN1-xTB provides a similar profile with a slightly deeper PMF well and shorter interaction range (see Section S13). The range of the flat-bottom region of the external potential is indicated by different colors.

**Figure 6:**
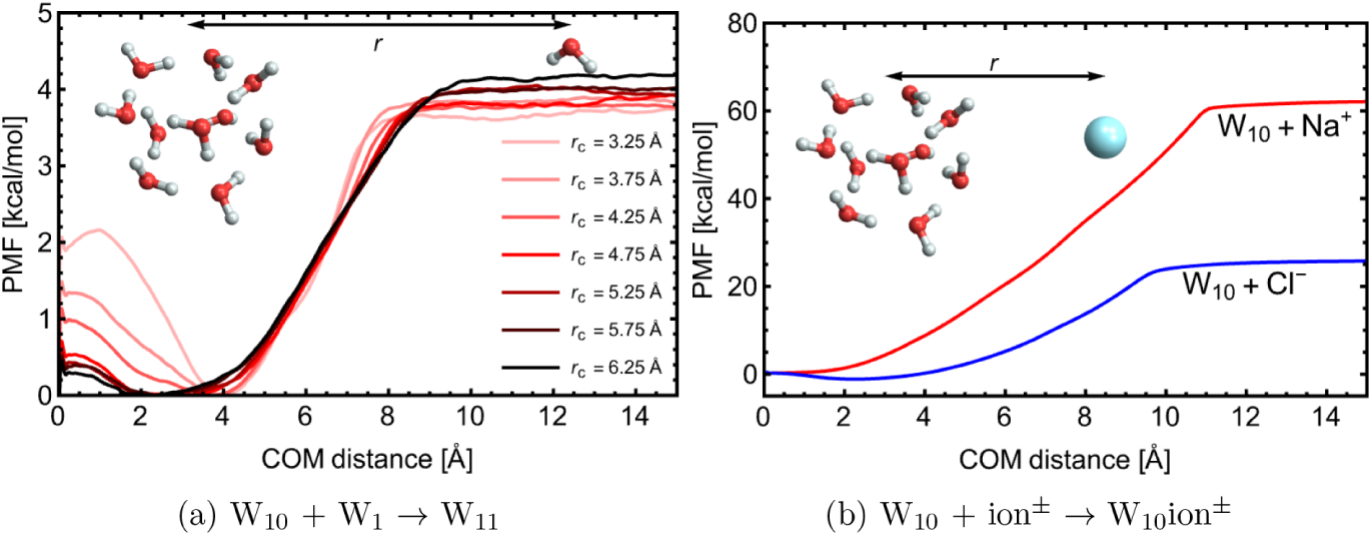
Free energy profiles (PMF) for uptake of a) water and b) Na^+^/Cl^–^ ion onto a 10-molecular water cluster. a) has been obtained at the GFN-FF level of theory and b) at GFN1-xTB.

As in Section S13, a too narrow flat-bottom potential range can compress the cluster, preventing the incoming water molecule from entering, and thereby reducing the interaction free energy. Conversely, if the range is too wide, evaporation is not effectively prevented, and the cluster may adopt an unphysical, elongated shape to accommodate the incoming water molecule. A well-chosen range prevents unwanted evaporation, without significantly perturbing the cluster and allowing the incoming water molecule to enter it. To balance these effects, we applied a cutoff of 4.75 Å, which effectively prevents evaporation while avoiding artificial compression.

Notably, the water-centered configuration (*r* = 0) in Figure 6a has a slightly higher free energy (∼0.4 kcal/mol) than the PMF minimum. This may indicate that placing a water molecule at the cluster center does not provide the most stable configuration, but it could also result from errors due to numerical sensitivity at short distances (see Section S6) and the poorly defined distribution of COM distances below 0.5 Å.

Figure 6b presents the PMF of sodium (Na^+^) and chloride (Cl^–^) ion uptake onto the constrained water cluster calculated at the GFN1-xTB level. The sodium ion has a binding free energy of –57.78 kcal/mol, significantly deeper than that of the chloride ion at –24.15 kcal/mol. This difference arises from the small size and high charge density of Na^+^, which leads to stronger interactions with water molecules. In contrast, the Cl^–^ has a lower charge density and higher polarizability, resulting in weaker binding. Moreover, Cl^–^ preferentially adheres to the surface, as indicated by a ∼1.1 kcal/mol dip around 2.25 Å. This illustrates the ion-specific preference of surface interactions, a phenomenon explored in multiple previous studies. From the experimental point of view, the existence of a water/vapor charge preference remains debated.^117^ According to Hartkamp et al.,^118^ this skepticism primarily stems from misinterpretation of the experimental techniques and error propagation in the analysis, which necessitates computational modeling to correctly interpret the raw data. Indeed, numerous theoretical^119^ and computational studies suggest a sign-preference at solid–electrolyte interfaces. These include force-field MD simulations,^120^ ab initio MD simulations^121,122^ force-field US simulations^123,124^ and thermodynamics quantum chemistry calculations.^125–127^ However, classical force field methods are often questioned due to concerns about their accuracy and their strong reliance on the choice of empirical potential. Conversely, quantum chemistry approaches combined with statistical thermodynamics may be criticized for neglecting entropic contributions. Our semiempirical US simulations help address these limitations by providing a computationally efficient yet thermodynamically informed methodology.

While we observed similar results between GFN1-xTB and GFN-FF, employing US with a more accurate QC level may be necessary for other systems, particularly when reactions between the cluster and the colliding molecule occur. For the systems presented here, fully semiempirical simulations were computationally feasible; however, this approach may become too demanding for larger clusters. In such cases, a QM/MM approach^128^ can be adopted, where the colliding molecule and, eventually, nearby cluster molecules are treated with QC methods, while the remainder of the system is described using molecular mechanics (MM). Alternatively, machine-learning (ML) techniques can replicate high levels of theory at a computational cost comparable to, or even lower than, that of semiempirical methods.

### 3.4 Machine-Learning-Enhanced Umbrella Sampling

In previous sections, we employed US at the XTB level. In this section, we validate our data generation and ML-enhanced US methodology for its ability to accurately reproduce a binding free energy at US^XTB^. Then, we apply the methodology with ML trained at an even higher level to atmospherically relevant systems.

#### 3.4.1 Validation of the Methodology

To validate our ML methodology for data preparation, curation, training, and application, we trained ML models at the XTB level and compared the PMFs and binding free energies from US simulations using these models (US^ML‑XTB^) with those obtained directly from XTB simulations (US^XTB^). Several possible sources of error can arise when comparing US^ML‑XTB^ and US^XTB^ results: (1) discrepancies between the QC programs. (2) the statistical uncertainty inherent in US, and (3) the accuracy of NN in reproducing the XTB energies and forces. The former issue is unfortunate, as we used the *tblite* program throughout the previous sections, whereas the NN was trained on energies and forces obtained with the native *xtb* program. Fortunately, differences in parametrization and algorithms between these implementations are minimal, with energy discrepancies <0.01 kcal/mol across all systems. Moreover, as shown in Section S8, the statistical uncertainty in the US simulations is also small (<0.3 kcal/mol). Thus, among these possible sources, the most critical is the accuracy of the NN in reproducing the forces of the reference method.

As an initial test, we applied ML to water dimerization. The NN model was trained only on ∼3k structures generated from short US simulations at 300 K (see Section 2.4), resulting in a validation error on the forces of 0.04 kcal/mol/Å. Using the trained NN potential in US simulations, we obtained a binding free energy of Δ*G* = 0.81 kcal/mol, which is satisfactorily close to the 0.90 kcal/mol obtained directly from US^XTB^ in Section 3.2.2.

As a second test, we trained an ML model for formic acid dimerization, also trained on only ∼3k structures. This resulted in significant errors in binding free energies (tens of kcal/mol). Hence, we trained a new ML model using 10.3k structures generated from all data generation schemes (US at 300 and 500 K, and ADGA), with a cutoff radius of 5 Å. The resulting US^ML‑XTB^ simulation produced a binding free energy of Δ*G* = −5.2(5) kcal/mol, in excellent agreement with the −5.4(8) kcal/mol obtained from US^XTB^.

As a final test, we applied ML to sulfuric acid dimerization, focusing on the effect of including data from different data generation schemes (see Section 2.4) and varying the atomic environment cutoff radius. Table 2 summarizes the different combinations of data sets used to train the ML models and the resulting Δ*G* values. The corresponding PMFs are shown in Figure 7. The PMFs obtained from ML models trained on the different data set combinations show deviations comparable to the numerical uncertainties inherent to US simulations. The resulting binding free energies are consistent with the reference value of Δ*G* = −4.1(5) kcal/mol obtained from US^XTB^. Note that the structures generated from US simulations at large intermolecular distances (e.g., 10 Å) effectively correspond to two noninteracting monomers. As a result, some degree of monomer sampling is included. However, ADGA systematically performs normal mode sampling to construct the potential energy surface with the least uncertainty.

**Figure 7:**
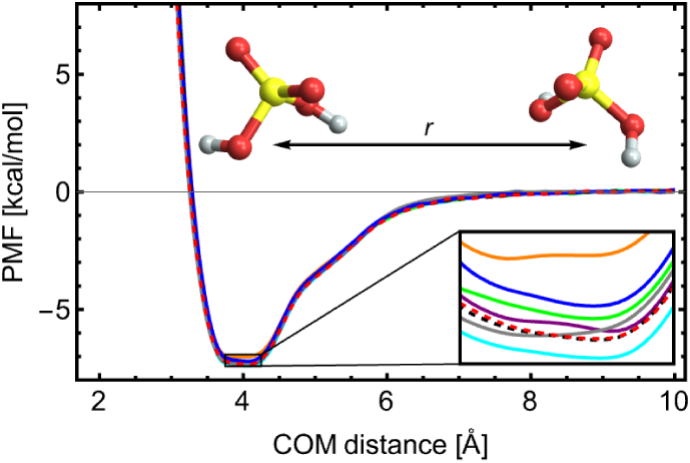
PMF of sulfuric acid dimerization obtained at GFN1-xTB level of theory using the Langevin/CSVR thermostats (black/red dashed lines). The solid lines correspond to PMFs obtained via NNs trained on the same level with data sets presented in Table 2: I = blue, II = orange, III = purple, IV = gray, V = green, VI = cyan.

**Table 2:** Overview of Data Sets Used, Cutoff Radius, and Resulting Binding Free Energies for SA_1_+SA_1_ Collision^a^

	**Included data sets**				**Cutoff**	**Δ** *G*
	∼3.3k from 100k step US at 300 K	∼6.5k from 100k step US at 500 K	∼1.6k from ADGA *trans*-SA	∼1.5k from ADGA *cis*-SA	[Å]	from US^ML‑XTB^ [kcal/mol]
I	×				10	–4.0(0)
II		×			10	–3.8(7)
III	×	×			10	–4.1(2)
IV	×	×			5	–4.1(8)
V	×	×	×		10	–4.1(2)
VI	×	×	×	×	10	–4.2(3)

a The target US^XTB^ has binding free energy of −4.1(5) kcal/mol.

Decreasing the atomic environment cutoff radius accelerates NN training. However, if the cutoff is too small, the ML model may fail to accurately capture long-range interactions. In the case of sulfuric acid dimerization, when the COM distance between the molecules is 7 Å, the closest atoms are separated by approximately 5 Å. The mean force at this COM distance is negligible. Therefore, a 5 Å cutoff radius is large enough to accurately capture the relevant interactions.

These tests demonstrate that generating data from US simulations at the target and elevated temperatures and normal mode sampling provides sufficient sampling for training sets that adequately reproduce the reference level of theory. Nonetheless, future studies should aim to reduce the size of the training set and the complexity of the NN, as the current NN requires substantial time for both training and evaluation. For example, for water dimerization, the NN potential is up to four times slower than direct XTB calculations. However, the advantages of ML become more apparent for larger systems: for US simulations of sulfuric acid dimerization, the ML model requires only ∼70% of the computational time needed for XTB. This speedup is expected to increase further with system size. Note that this comparison excludes the time required for data generation and model training. Most importantly, NN models enable US simulations at levels of theory that would be infeasible to employ directly.

#### 3.4.2 Machine Learning Application

To apply our ML methodology, we performed ML-enhanced US simulations of two systems: water dimerization, for which experimental data are available, and the atmospherically relevant binding of sulfuric acid and ammonia, for which currently no experimental data exist. The training data sets comprised ∼6.3k structures for the W_1_+W_1_ system and ∼16.4k structures for the SA_1_+AM_1_ system.

We trained ML models at both the DFT and DLPNO levels of theory and compared the resulting binding free energies from ML-enhanced US simulations to those obtained directly from standard QC calculations at the same levels of theory. Figure 8 shows the binding free energy predictions for the W_1_+W_1_ and SA_1_+AM_1_ systems. For the W_1_+W_1_ system, the US^ML‑DLPNO^ binding free energy is approximately 0.5 kcal/mol lower than the experimental value. Assuming that CCSD­(T) provides the most accurate potential energy surface, we would expect US^ML‑DLPNO^ simulations to closely match experimental results. The domain-based local pair natural orbital approximation makes CCSD­(T)-level calculations feasible for larger systems. However, the absence of analytical gradients in DLPNO–CCSD­(T_0_) necessitates the use of numerical gradients, which significantly slows down data preparation for ML training. Both the numerical gradients and the high sensitivity of DLPNO–CCSD­(T_0_) to the numerical PNO thresholds reduce the accuracy of interatomic forces. As a result, large validation errors on the forces (0.23 kcal/mol/Å for W_1_+W_1_ and 0.29 kcal/mol/Å for SA_1_+AM_1_) indicate that training ML models on inaccurate interatomic forces is more challenging and more training would be required for better accuracy. For comparison, ML models trained at the DFT level achieve much lower force errors of 0.05 kcal/mol/Å for both systems. This reduced ML model accuracy at the DLPNO level likely contributes to the discrepancy observed in water dimerization between US^DLPNO^ and experiments.^95–99^

**Figure 8:**
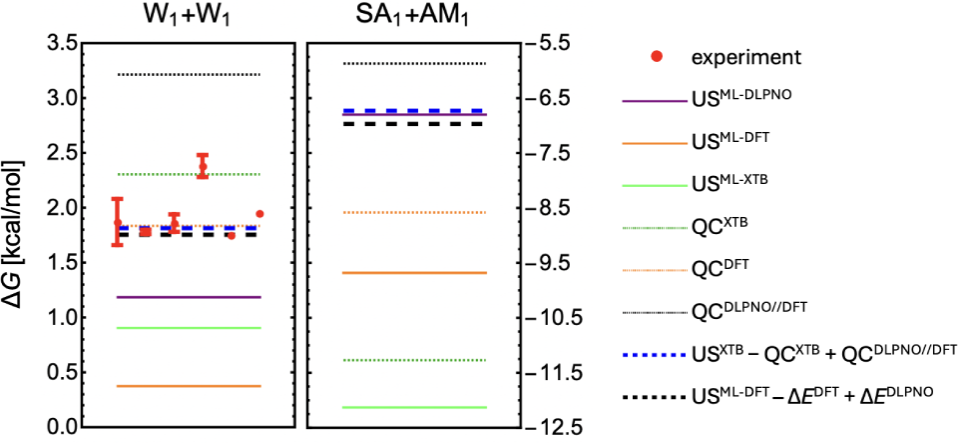
Binding free energy obtained through US and QC-based traditional thermodynamics at different methods for water (W) dimerization and binding of sulfuric acid (SA) with ammonia (AM). Δ*E* is the electronic binding energy.

Because the DLPNO training data was generated by performing DLPNO gradient calculations on structures initially obtained from the initial XTB structural screening simulations. Hence, the errors could also stem from differences between the energy surfaces at the XTB and DLPNO levels. To address this, we generated an additional 3k structures from the initial US^DLPNO^ simulation of W_1_+W_1_, computed their DLPNO gradients, and included them in the training set. While this reduced the ML validation error, it had a negligible impact on the predicted binding free energy (∼0.1 kcal/mol difference).

Figure 8 also shows that adding the previously neglected entropic effects obtained at XTB (US^XTB^–QC^XTB^) to the high level DLPNO//DFT free energies or thermal contributions obtained at DFT (US^ML‑DFT^–ΔE^DFT^) to the high-level DLPNO electronic binding energies accurately reproduces the experimental water dimerization free energies (blue and black dashed lines). Applying this reasoning to SA_1_+AM_1_ binding suggests that the binding free energy may be ∼1 kcal/mol lower than predicted by the standard QC approach. This would have important implications for NPF modeling, as a 1 kcal/mol decrease in binding free energy roughly corresponds to an order-of-magnitude reduction in cluster evaporation rates, thereby increasing cluster stability and enhancing their contribution to NPF.

In future studies, it will be important to identify reliable methods that provide analytical gradients, such as RI-MP2. Additionally, generating only the essential portion of training data—and thereby minimizing the number of energy and gradient evaluations at the desired high level of theory—would greatly improve the efficiency of the ML methodology.

## 4 Conclusions

As electronic energies are being calculated with increasing accuracy for an expanding range of cluster sizes and compositions, the statistical thermodynamic treatment of local and global anharmonic contributions must likewise become more accurate. To this end, we combined the enhanced-sampling molecular dynamics technique, umbrella sampling, with a quantum Hamiltonian to capture the entropic contributions typically missing from standard quantum chemistry approaches. Umbrella sampling was performed along the evaporation coordinate of a molecule from a cluster, providing the potential of mean force along this coordinate. Binding Gibbs free energies calculated from the potential of mean force include entropic contributions. These entropic contributions were calculated at the semiempirical GFN1-xTB level of theory for a range of atmospherically relevant systems and used as corrections to standard DLPNO–CCSD­(T_0_)//ωB97X-D binding free energies. On average, this resulted in improved agreement with experimental data, reducing the mean absolute error from 1.38 to 0.74 kcal/mol across the studied systems. We also showed that the shape of the potential of mean force provides insights into how molecules are incorporated into clusters, including their preferred location and the presence of multiple cluster–molecule configurations along the evaporation coordinate.

Traditional quantum chemistry approaches often rely on heavy approximations or reduced levels of theory to make configurational sampling applicable for larger clusters. As a result, the accuracy of binding free energies obtained from these methods can be difficult to assess. In contrast, our approach directly captures entropic effects via umbrella sampling and provides a path to more reliable free energy predictions—even for larger systems—if the underlying potential energy surface (i.e., computational method) is accurate.

While well-converged umbrella sampling simulations are computationally intensive, their cost can be greatly reduced using machine learning interatomic potentials trained at a high level of theory. We demonstrated that the polarizable atom interaction neural network (PaiNN), trained on configurations from umbrella sampling simulations, accurately reproduces reference-level results. With these potentials, the binding free energy of a sulfuric acid–ammonia cluster was found to be up to 1 kcal/mol lower than that predicted by standard DLPNO–CCSD­(T_0_)/aug-cc-pVTZ//ωB97X-D/6–31++G­(d,p) methods, highlighting the importance of entropic contributions.

This work introduces a new and scalable methodology for computing binding free energies that goes beyond the limitations of conventional approaches by including entropic effects explicitly and enabling systematic application to larger clusters. Future work should focus on expanding the range of systems studied, improving training set generation for machine learning, and applying the trained potentials to other processes where entropic effects are expected to play a key role, such as nucleation and cluster-mediated reactions.

## Supplementary Material



## Notes

S1: Free energy sensitivity to bound state choice; S2: Quantum corrections; S3: Technical details for US simulations; S4: Machine learning technical details; S5: Normal mode sampling; S6: Blank test; S7: Two atom collisions; S8: Thermostat; S9: Preventing unwanted evaporations; S10: Free energy convergence; S11: Binding free energy values; S12: PMFs of all studied systems; S13: Free energy profile of W_10_+W_1_; (PDF)
