# Supplementary material for: Thermodynamics of Molecular Binding and Clustering in the Atmosphere Revealed through Conventional and ML-Enhanced Umbrella Sampling
Source: ACS Omega. 2025 Aug 18;10(34):39148–61. doi: 10.1021/acsomega.5c05634 (PMC12409561; doi:10.1021/acsomega.5c05634)
Supplement: Supplementary file 1 [file ao5c05634_si_001.pdf]

Supporting Information
for
Thermodynamics of molecular binding and clustering in the atmosphere revealed through conventional and ML-enhanced umbrella sampling

Jakub Kubečka,*,† Yosef Knattrup,† Georg Baadsgaard Trolle,† Bernhard Reischl,‡ August Smart Lykke-Møller,† Jonas Elm,† and Ivo Neefjes†

†*Aarhus University, Department of Chemistry, Langelandsgade 140, Aarhus, DK 8000*

‡*University of Helsinki, INAR/Physics P.O.Box 64 Helsinki, FI 00014*

E-mail: ja-kub-ecka@chem.au.dk

Phone: +420 724946622

S1 Free energy sensitivity to bound state choice

The integral in Eq. (7) depends on how the bound state is defined. For the binding of two gas-phase species in the free molecular regime, the bound state can be identified as all center-of-mass (COM) distances where significant interactions occur, i.e., within the well of the potential of mean force (PMF). Typically, there is a single overall well, which may contain multiple local minima.

The integrand in Eq. (7) is a Boltzmann factor over the projection of the Helmholtz free energy $F(r)$ along the COM distance r. $F(r)$ consists of the PMF $w(r)$ and an entropic term $-k_\mathrm{B}T\ln(r^2)$, which accounts for the increase in the number of microstates available to the system as r increases. $F(r)$, $w(r)$, and $-k_\mathrm{B}T\ln(r^2)$ are shown on the left side of Fig. S1 for the case where $w(r)$ follows a Lennard-Jones (LJ) 12–6 potential given by:

$$w(r) = 4\varepsilon\left[\left(\frac{\sigma}{r}\right)^{12} - \left(\frac{\sigma}{r}\right)^{6}\right] \tag{S1}$$

where ε and σ are the LJ energy and length parameters, respectively.

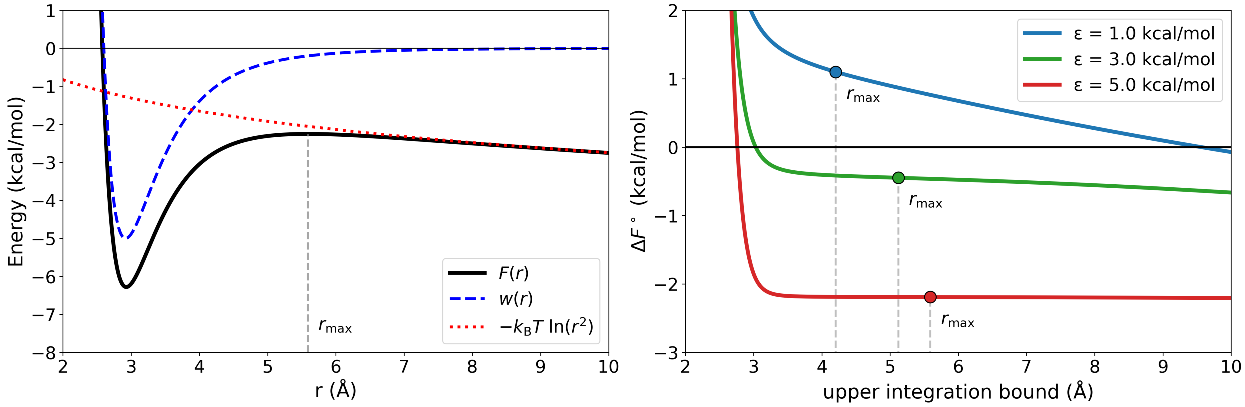

Figure S1: Left: An example of the Helmholtz free energy profile $F(r) = w(r) - k_\mathrm{B}T\ln(r^2)$ (black line), the potential of mean force $w(r)$ (blue dashed line), and the entropic term $-k_\mathrm{B}T\ln(r^2)$ (red dotted line) along the center-of-mass distance r for two particles interacting via a Lennard-Jones potential with $\varepsilon = 5.0$ kcal/mol and $\sigma = 2.6$ Å. Right: ΔF as a function of the upper bound of the integral in Eq. (8) when $w(r)$ is a Lennard-Jones potential with $\sigma = 2.6$ Å and ε values of 1.0 (blue), 3.0 (green), and 5.0 kcal/mol (red). The position of the local maximum of $F(r)$ corresponding to each Lennard-Jones potential is also indicated.

The well of the PMF is typically located at low r, resulting in a peak in the integrand. However, at large r, the entropic term becomes increasingly significant. When defining the bound state, integration should be performed over the well while minimizing contributions from the entropic term outside the well. A reasonable upper bound is the position of the maximum of $F(r) = w(r) - k_\mathrm{B}T\ln(r^2)$, which marks the point where the entropic term starts to become dominant in shaping the free energy profile projected along r.

The relative importance of the PMF $w(r)$ and the entropic term depends on the well depth, which in turn is influenced by the strength of the intermolecular interactions. To illustrate this dependence, Figure S1 also shows ΔF as a function of the upper bound of the integral in Eq. (8) for a PMF described by the Lennard-Jones (LJ) 12–6 potential with $\sigma = 2.6$ Å and varying ε at 298.15 K. For $\varepsilon = 5.0$ kcal/mol, the difference between integrating up to $r_\mathrm{max} = 5.6$ Å or 10 Å is only 0.01 kcal/mol. However, for $\varepsilon = 1.0$ kcal/mol, the difference between integrating up to $r_\mathrm{max} = 4.2$ Å or 10 Å is 1.17 kcal/mol. Thus, the definition of the bound state is particularly important for weakly interacting systems.

The position of the maximum of $F(r)$ is an unambiguous upper bound, ensuring that the integral only runs over the well.

S2 Quantum corrections

Here, we examine the difference in free energy of a potential well treated through classical and quantum chemistry. As a first approximation, the potential well can be characterized as harmonic. The Helmholtz free energy F_ho of a harmonic oscillator with eigenfrequency ω is given by[1]

$$F_\mathrm{ho} = \frac{1}{\beta} \cdot \ln(\beta\hbar\omega). \tag{S2}$$

On the other hand, the Helmholtz free energy of a quantum oscillator (qo) is[2]

$$F_\mathrm{qo} = \frac{1}{2}\hbar\omega + \frac{1}{\beta} \cdot \ln\left(1 - e^{-\beta\hbar\omega}\right). \tag{S3}$$

The Helmholtz, as well as the Gibbs, binding free energy quantum correction is equal to the difference between the free energies of the quantum and classical harmonic oscillators:

$$\Delta G_{\mathrm{corr}} = \Delta F_{\mathrm{corr}} = F_{\mathrm{qo}} - F_{\mathrm{ho}} = \frac{1}{2}\hbar\omega + \frac{1}{\beta} \cdot \ln\Big((1 - e^{-\beta\hbar\omega})/(\beta\hbar\omega)\Big) \tag{S4}$$

Practically, we solve the Schrödinger equation for the 1-dimensional PMF well without the harmonic approximation, using the reduced mass of the two binding molecules. We then take the zero-point energy (ZPE) (the first energy level in Fig. 1 of the main paper) and insert it (i.e., $\hbar\omega = 2 \cdot \mathrm{ZPE}$) into Eq. S4 to obtain the free energy correction. Figure S2 shows the quantum correction to the free energy. In this work, the ZPE ranges from 0 to 0.4 kcal/mol.

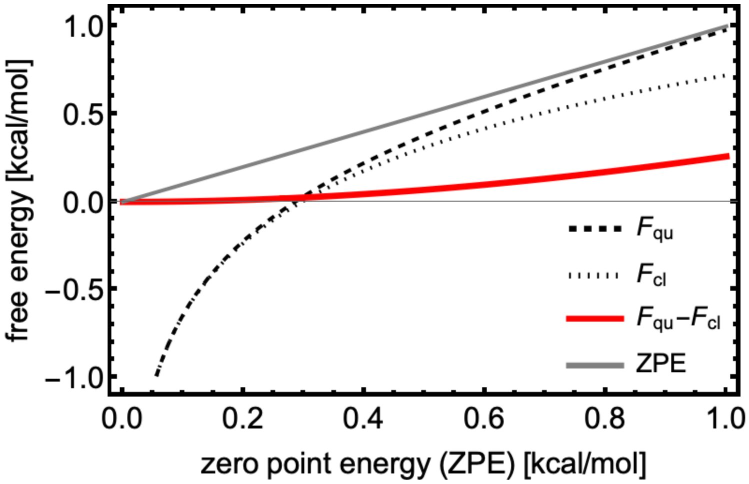

Figure S2: Free energy correction (red) between quantum (dashed-black) and classical (dotted-black) harmonic oscillators at room temperature according to zero point energy (ZPE) of this well. The graph shows that at this temperature, the correction is negligible.

The correction is most significant for strongly bound clusters and collisions involving ions, as both exhibit narrower PMF wells. However, while we include the quantum correction in this study, its overall impact is negligible (< 0.03 kcal/mol). When the colliding species are separated or for potential energy surface (PES) minima along other dimensions, these corrections are not needed, as they are typically well-propagated in the molecular dynamics simulations used to obtain the PMF. We again note that this correction becomes significant at low temperatures. As an illustrative example, the QC correction for water dimerization

is 0.013 kcal/mol at 300 K and already 0.12 kcal/mol at 233 K.

S3 Technical details for US simulations

Here, we elaborate on technical details of the umbrella sampling (US) simulations.

Kästner and Thiel[3] emphasized that k_{bias} must be greater than $-\partial^2 F(r)/\partial r^2$ at every point along r to minimize systematic errors. However, a too large k_{bias} requires long simulations to ensure sufficient overlap between windows. To balance accuracy and efficiency, k_{bias} is typically chosen to be 2–5 times greater than $-\min(\partial^2 F(r)/\partial r^2)$. Since the free energy profile is a priori unknown, selecting an appropriate k_{bias} generally involves a trial-and-error approach. In this study, we used a fixed value of 100 kcal/mol/Å for all simulations, which we later confirmed to be adequate for all studied systems by analyzing the resulting PMFs. However, if better efficiency, i.e., shorter simulations, are necessary, multiple systems and collective variable (CV) ranges could be sampled with a lower bias.

To further minimize systematic errors, the window spacing Δr_{bias} should be smaller than $3/\sqrt{\beta k_{\text{bias}}}$ (i.e., < 0.42 Å in our case).[3] In our simulations, we use $\Delta r_{\text{bias}} = 0.2$ Å. The number of windows depends on the system and CV under study. While using short simulations over many windows rather than long simulations over a few windows has been shown to reduce statistical error (and is better for parallelization),[4,5] simulations that are too short may fail to adequately sample the system in unconstrained directions, particularly when significant barriers exist in these directions.

All simulations were performed in the canonical (NVT) ensemble, with a timestep of 1 fs, and either with the Langevin[6] or canonical sampling through velocity rescaling (CSVR[7]) thermostat. We tested the validity of this timestep on the smallest collision system (W_1+W_1). The vibration due to the bias potential with $k_{\text{bias}} = 100$ kcal/mol/Å has a period of ~92 fs. This period will increase with system size. Hence, hydrogen vibrations (up to 4000 cm^{-1}, i.e., ~8 fs period) are the fastest vibrations, requiring a timestep below 4 fs.

Thus, our 1 fs timestep is sufficiently small.

Our US workflow involves three different steps. First, we performed an unbiased molecular dynamics (MD) simulation of each colliding species separately for 100,000 timesteps. These simulations were used for configurational sampling (CS) to obtain several reasonable initial structures for the US simulations. Initial geometries for these CS simulations were generated using the ABCluster program.[8,9] At the start of the CS simulation, initial velocities for each atom are randomly drawn according to the Maxwell–Boltzmann distribution at 300 K. Based on the work of Halonen et al.,[10] who studied the role and effect of thermostats during system equilibration and the actual run, we used the Langevin thermostat with a coupling constant of 0.01 fs^{-1} to reach and maintain the target temperature of 300 K.

We estimate suitable equilibration durations and output frequencies by examining the autocorrelation of various properties in equilibration runs of water clusters up to W_{128}, using the GFN-FF force field. These properties included the radius of gyration, total temperature (along with its translational, vibrational, and rotational components), and total energy and its individual contributions. The results are presented in detail in Sec. S3.1. Because the autocorrelation of the temperature partitions is primarily driven by the thermostat coupling constant, their autocorrelation times are relatively short (approximately 100 fs). In contrast, the autocorrelation times of the radius of gyration and total/potential energy range from roughly 200 fs for small clusters (W_{2-4}) to around 700 ps for the larger ones (W_{32-128}). As a compromise between reducing correlation and managing computational cost, we chose to save the structures and thermodynamic properties every 250 timesteps (i.e., every 250 fs). Based on the Mann–Kendall test applied to the decorrelated time series, equilibration is typically reached within 2 to 15 ps. Consequently, we equilibrated all systems for 100 ps and used the final 25 ps to extract initial configurations for US. With outputs saved every 250 fs, this yields 100 structures per system.

In the second step of our US workflow, we perform US equilibrium simulations. For each collision system, the two colliding species are placed at a COM distance corresponding

to the current US window. The initial structure of both species is taken from the above CS simulations. At the start of the equilibration simulation, the velocities of the atoms are reinitialized according to the Maxwell–Boltzmann distribution at 300 K. The equilibration is performed for 100 ps using the Langevin thermostat. For windows centered at low r values, the initial placement of the two structures can result in them overlapping. In this case, we place the structures further apart and linearly increase the US harmonic potential within the first 1,000 simulation steps so the system can find an appropriate structure for the specific window on its own.

The last step comprises the actual US production run. In the production run, we either used the Langevin thermostat or switched to the CSVR thermostat. During the production run, structures are saved every 250 timesteps (i.e., every 250 fs). In Sec. S3.2, we show that the autocorrelation times for the COM distance are similar for W_1+W_1 and SA_1+SA_1 binding, as well as between GFN-FF and GFN1-xTB. However, they differ between the Langevin thermostat ($<$ 200 fs) and the CSVR thermostat (several ps). The Langevin thermostat functions by mimicking stochastic local collisions with a carrier gas. As such, it allows for fast thermal equilibration between all degrees of freedom even for gas-phase systems.[10] Therefore, a coupling constant of 0.01 fs^{-1} is sufficient for Langevin thermostats, but simulations with the CSVR thermostat, and especially those of small molecular systems, might possess a certain level of correlation (see paragraph above). Decreasing the output frequency while keeping the same number of samples would require longer simulations, which would come with extra computational costs. Nevertheless, as the actual runs are 0.5 ns long for each window, there is still enough (at least 500) uncorrelated data. Moreover, we combine the histogram distributions from 10 independent simulations for each window, which guarantees a well-sampled configurational space.

The accuracy of umbrella integration (UI) depends on the resolution of the integration grid, determined by the number of bins along the CV. While increasing the number of bins improves resolution, an excessively fine grid can lead to slow convergence. Typically, 200–400

bins are used for the full CV. In this work, we use a bin width of 0.02 Å, resulting in a total number of 250–1000 bins. For greater detail on WHAM and UI, see the review by Kästner.[5]

S3.1 Correlation and equilibration times

In this section, we investigate correlation and equilibration times. We performed 100 ps long MD simulations of 1–128-molecular water clusters at the GFN-FF level of theory while using the Langevin thermostat. To prevent evaporation, we used a flat bottom harmonic potential with a force constant of 5 kcal/mol/Å^2 and a flat radius threshold corresponding to 110 % of the cluster's equilibrium radius of gyration. We used an integration timestep of 1 fs and dumped system properties every 10 fs. The first 20 ps were removed from the analysis as we estimated that equilibration will take less than 20 ps, which we later confirm with Mann–Kendall test for no upward/downward trend (see below). Figure S3 represents autocorrelation times (i.e., when autocorrelation function drops below $1/e$) for system properties: radius of gyration (R_g), total temperature (T_total), vibrational temperature (T_vib), rotational temperature (T_rot), translational temperature (T_transl), total energy (E_total), kinetic energy (E_kin), and potential energy (E_pot). The kinetic energy and total temperature overlap as they are proportional to each other. All temperatures have autocorrelation times around 100 fs as they are coupled to the Langevin thermostat with a coupling constant of 0.01 fs^{-1}. The potential/total energy and radius of gyration have the longest autocorrelation time which increases with system size. Large water clusters require output intervals of more than $\sim$800 fs for uncorrelated data.

We have additionally taken the entire 100 ps long simulation and separated the data into frames of simulation length corresponding to the correlation times of potential energy, one of the slowest variables to decorrelate. For each frame, we calculated the mean value of the potential energy and analyzed how many initial frames have to be removed to observe no trend in the frame means (Mann–Kendall test with 90 % confidence, i.e., α=0.1). The removed simulation data length corresponds to equilibration times. Figure S4 shows that

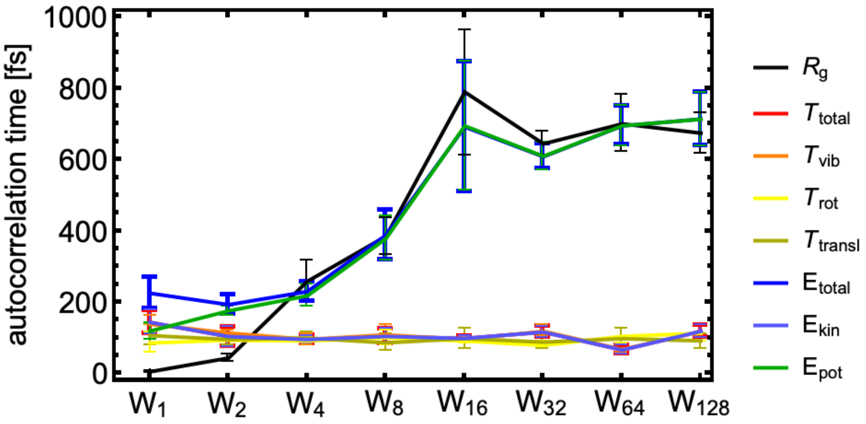

Figure S3: Correlation times of various properties for water clusters of various sizes.

because we started with reasonable water cluster structures and initiated the velocities according to the Maxwell–Boltzmann distribution at 300 K, the equilibration times are quite short, not exceeding 20 ps. Since we use a much larger duration of 100 ps for equilibration, we can confidently say that all our systems are well equilibrated.

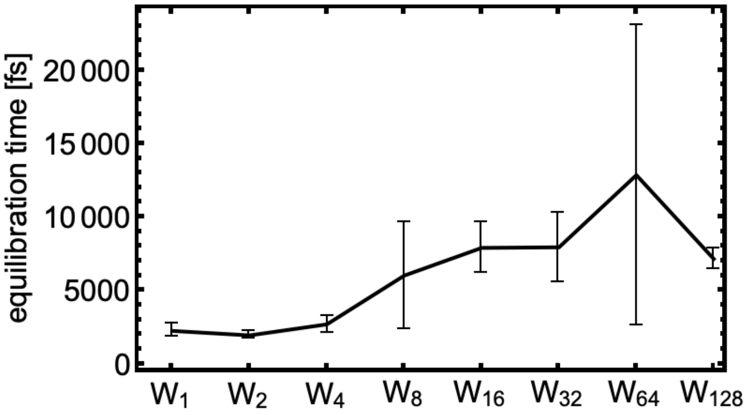

Figure S4: Equilibration times of water clusters of various sizes.

S3.2 Autocorrelation function of COM distance

In the previous section, we show the autocorrelation time of various properties of a single species. Here, we study the species pairs constrained with the bias potential during the

umbrella sampling simulations as we want to examine the autocorrelation times of the COM distance. Hence, we performed short 100 ps US simulations for water (W_1+W_1) and sulfuric acid (SA_1+SA_1) dimerization at either the GFN-FF or GFN1-xTB levels of theory and either with Langevin or CSVR thermostats. Figure S5 shows the autocorrelation function (ACF) of the COM distance (d_{COM}) from 3 different windows corresponding to the equilibrium bias distance. There are small differences between the windows. However, the main difference can be observed between the thermostats. The Langevin thermostats better redistribute excessive energies between all degrees of freedom, making the data less correlated than for the CSVR thermostat.

S4 Machine learning technical details

In this work, we used the same neural network (NN) architecture as Kubečka et al.[11] with similar (except for batch size [BS] and cutoff) hyperparameters: atom basis (AB) = 128, interaction layers = 5, radial basis (RB) = 20, cutoff = 5–10 Å, learning rate (LR) = 0.0001 with no scheduler, 400 epochs with no early stopping, batch size (BS) = 8, AdamW optimizer,[12] 10 % training data used for validation, and a loss function weighted as 99 % mean squared error (MSE) of all force components and 1 % MSE of energies.

Machine-learning potentials (MLPs) are functions that assign specific labels, such as energy (e.g., electronic energy, E_{el}), to a given molecular geometry. However, absolute energies can exhibit substantial systematic errors due to inconsistencies in reference states. Therefore, it is advantageous to utilize alternative reference states, leading to labels such as atomization energies ($\Delta_{\mathrm{a}}E_{\mathrm{el}}$) or, eventually, in the case of clusters, binding energies ($\Delta_{\mathrm{b}}E_{\mathrm{el}}$). Fitting relative energy differences offers several benefits, including reducing the already-mentioned systematic errors, improved transferability to other systems, and a narrower label range, which makes the learning process for ML models easier. The cost for this step is minimal as obtaining energies of atoms or eventually clusters' monomers is cheap. Moreover, this

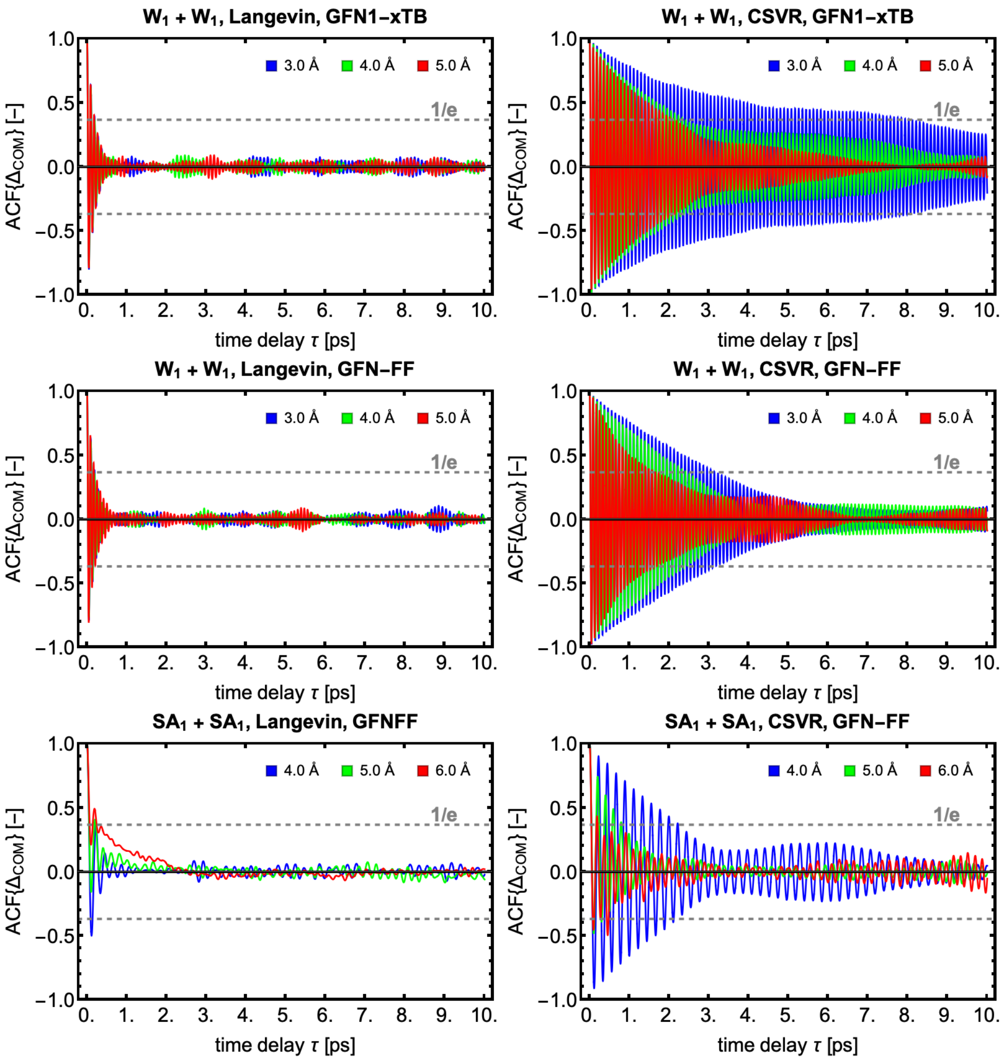

Figure S5: Autocorrelation function of COM distance. The period of the harmonic oscillator can also be visible in the autocorrelation function as well as the extent of the autocorrelation.

step can even be bypassed by assigning arbitrary reference energies to artificial atoms. For machine learning model training, this becomes especially beneficial when the artificial atomization energies exhibit minimal spread, which can be achieved by minimizing the following loss function

$$\mathcal{L} = \sum_{i}^{\text{train data}} \Delta_{\mathrm{a}} E_{\mathrm{el},i} = \sum_{i}^{\text{train data}} \Big(E_{\mathrm{el},i} - \sum_{j}^{\text{atoms in } i} E_{\mathrm{el},j}^{\text{artificial}} \Big), \tag{S5}$$

where $E_{\mathrm{el}}^{\text{artificial}}$ are the fittable artificial electronic energies of typically a few present atoms in the training data (e.g., $j \in$ [C, H, O, S,...]).

S5 Normal mode sampling

The ADGA[13] is an iterative method that automatically samples the single points required to construct a PES which includes n-mode couplings, where n can be defined on input. Given a reference structure (typically a minimum), normal modes are constructed and then some initial points are selected to construct an initial 1-mode PES. These points are chosen as the reference structure and the points along each normal coordinate corresponding to the n-th turning point of the quantum mechanical harmonic oscillator, where n can be given on input. This gives an initial $1 + 2M$ single points where M is the number of normal modes. Using these single points, a PES is fitted and a vibrational density is computed using vibrational self-consistent field theory.[14,15] The vibrational density is then used to compute an energy-like quantity in each interval between neighboring points along each normal mode. The single points, which are the midpoints between two neighboring points, are then computed and a new PES is constructed. Using this new PES, the energy-like quantity is then reevaluated and its change is compared to a number of convergence criteria. If the change in this quantity is sufficiently small, the interval is considered converged, and no further subdivision takes place. Otherwise, more single points are added iteratively until a 1-mode PES of sufficient quality has been constructed. This procedure is then repeated, constructing a 2-mode PES and so on until the desired n-mode PES has been obtained. An overview of the ADGA and

the prerequisite theory can be found in the review by Christiansen[16].

The ADGA is a method originally developed for use in PES construction for use in vibrational computations. However, the principle of selecting points that induce a large change in an energy-like quantity, a property of physical importance, is an active learning approach that samples the areas of a PES that are physically relevant. This means that the resulting database of single points is a suitable training set for ML models, something that has been used previously in conjunction with Gaussian process regression models.[17] In this work, we filter out high-energy monomer configurations ($>$ 25 kcal/mol above the lowest minimum) for efficient ML training.

S6 Non-interacting particles test

To assess the inherent error in our US methodology, we performed test US simulations of non-interacting particles. Ideally, this simulation should result in a flat PMF. However, due to numerical sensitivity at small COM distances (due to the logarithmic entropy correction) and ill-defined COM distribution close to 0 Å, the PMF has the largest errors at small COM distances. For COM distances greater than 0.5 Å, we can say that the error of our US approach is less than 0.1 kcal/mol. For the dimerization of two molecules or atoms, there will generally be significant repulsion at these small COM distances, and the PMF at these distances can therefore be neglected. This issue might be more profound in the case where the colliding molecule can enter the center of the cluster it collides with. In such cases, the inherent US errors associated with small COM distances should be considered. To conclude, for most cases presented in this paper, we estimate that the final binding free energy will be subject to an error no greater than 0.1 kcal/mol due to numerical sensitivities.

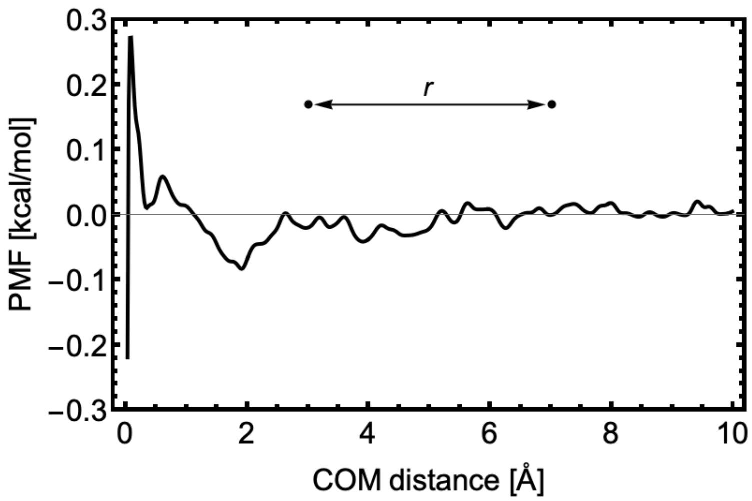

Figure S6: Potential of mean force (PMF) of two non-interacting atoms.

S7 Two atom collisions

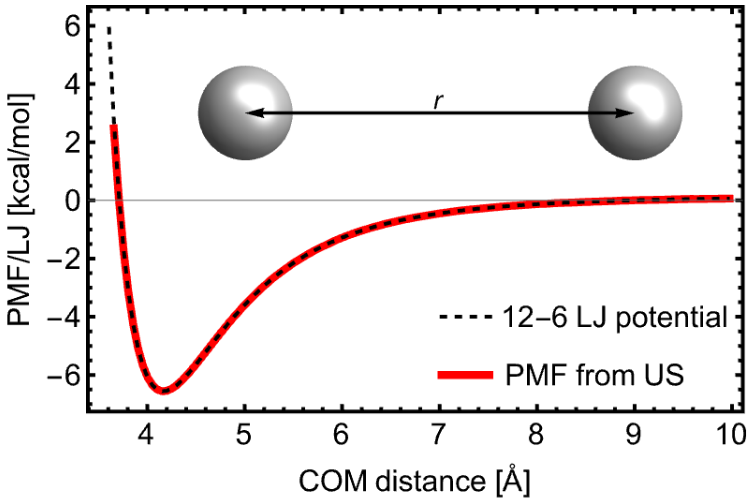

Figure S7: Potential of mean force (PMF) of two atoms interacting via LJ potential.

We employed US simulations with two atoms interacting through a Lennard-Jones potential with energy parameter $\varepsilon = 6.69$ kcal/mol and distance parameter $\sigma = 3.71$ Å. Figure S7 shows that the PMF obtained from US simulations directly coincides with the LJ potential. Therefore, direct integration of the LJ potential gives free energy of -3.10316 kcal/mol, which is the same as obtained from UI of the PMF: -3.10312 kcal/mol.

We have further examined argon–argon collision directly at the GFN1-xTB level of theory. The PMF (see Fig. S12) obtained from US simulations contains a significant amount of noise as the PMF well is only slightly deeper than the noise from US simulations (see Fig. S6). Nevertheless, even with this noise, integration of the PMF gives $\Delta G = 3.15$ kcal/mol, which is sufficiently close to $\Delta G = 3.14$ kcal/mol obtained from $\mathrm{QC}^{\mathrm{XTB}}$ (with no quasi-harmonic approximation applied). This further validates our algorithms and methodology.

S8 Thermostat

Thermostats adjust the system's kinetic energy to maintain a target temperature. This inherently alters the dynamics of the system, so it is important to choose an appropriate thermostat and a corresponding coupling/friction parameter. Halonen et al.[10] discussed unconstrained molecular dynamics (MD) simulations and suggest using the Langevin thermostat for equilibration and the CSVR thermostat for the actual sampling run, as CSVR better preserves the realistic vibrational motion of molecular systems. However, since the main task of US is to provide a correct spatial distribution, we are not concerned with unrealistic dynamics as long as relevant configurations are sampled along the CV. Therefore, both CSVR and Langevin thermostats should be suitable for the US production run.

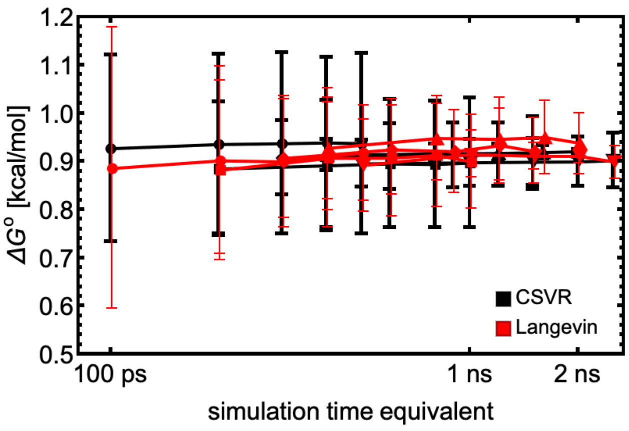

Figure S8: Convergence of water dimerization binding energy (ΔG) with simulation time equivalent, i.e., either running long simulations or combining multiple simulations when using Langevin or CSVR thermostat.

To test this hypothesis, we replaced the CSVR thermostat with the Langevin thermostat for the water dimerization simulations in Sec. S10. Figure S8 shows that both thermostats reach nearly the same result after combining simulations into 5 ns simulation time equivalent: 0.898 and 0.908 kcal/mol for CSVR and Langevin, respectively. While the error bars decrease more steadily for the Langevin thermostat (likely due to better exchange of kinetic energy between the available degrees of freedom), both thermostats provide similar results. We also examined the choice of CSVR/Langevin thermostat for sulfuric acid dimerization

(−4.152/−4.156 kcal/mol), formic acid dimerization (−5.481/−5.404 kcal/mol), dimethylamine and water binding (0.979/0.917 kcal/mol), and water addition to a sulfuric acid–dimethylamine cluster (−5.208/−5.060 kcal/mol), which together show that the difference is less than 0.15 kcal/mol for dimerizations. In this work, we primarily use the CSVR thermostat for US production runs, but for simulations with an external potential applied to one of the binding species, we used the Langevin thermostat instead, as detailed in Sec. S9.

S9 Preventing unwanted evaporations

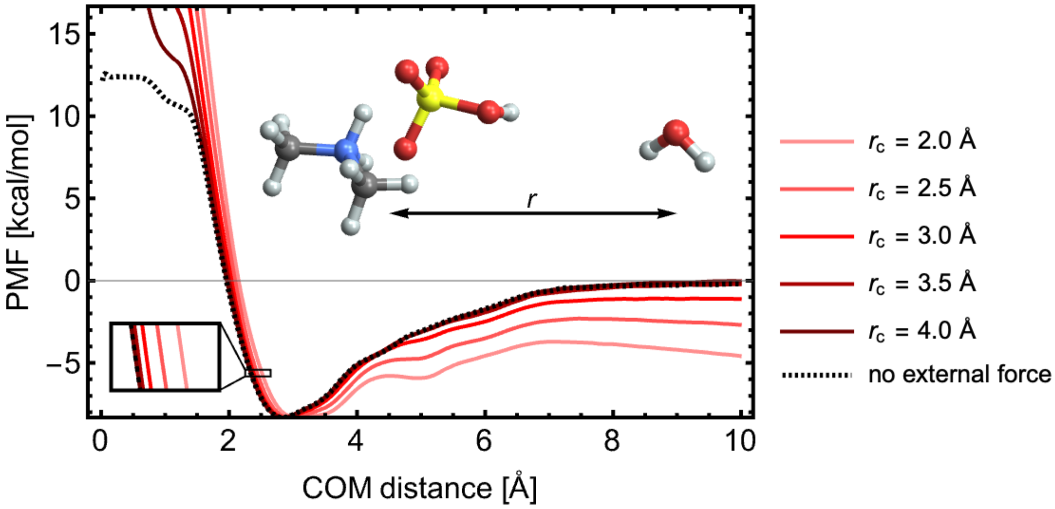

Figure S9: Potential of mean force (PMF) for clustering of SA_1DMA_1 and W_1. External force in the flat bottom harmonic potential, with cutoff COM radius specified in the legend, is applied on the SA_1DMA_1 to test whether its evaporation prevention affects the PMF calculations. Radius of gyration for SA_1DMA_1 in no-external-force simulation fluctuates in range 1.9–2.2 Å and the maximal atom-pair distance in range 6.0–7.3 Å.

Some clusters might be weakly bound and prone to evaporation during the US simulations. For instance, studying the evaporation of a specific water molecule from a water cluster can be complicated by the evaporation of other water molecules from the cluster. To prevent unwanted evaporations, we can apply an external potential to these clusters. We use a flat-bottom harmonic potential applied to each atom of the molecules, where we want

to suppress evaporation, in order to constrain the radius of the cluster:

$$V_{\mathrm{EF}}(\vec{r}) = k_{\mathrm{EF}}(|\vec{r} - \vec{r}_{\mathrm{COM}}| - r_{\mathrm{c}})^2 \cdot \mathrm{Heaviside}(|\vec{r} - \vec{r}_{\mathrm{COM}}| - r_{\mathrm{c}}), \tag{S6}$$

where $\vec{r}$ is the atomic position, $\vec{r}_{\mathrm{COM}}$ the cluster's COM position, r_{c} the flat bottom cut-off, and the Heaviside step function controls the change between the flat bottom and the harmonic potential.

To test the effect of the external potential on the dynamics of the system, we applied the flat-bottom harmonic potential with different r_{c} values to the SA_1DMA_1 cluster, which is stable and does not evaporate within our simulation times. We then performed US simulations of SA_1DMA_1 and W_1 binding. Figure S9 shows that PMFs corresponding to r_{c} values of 3.5 and 4.0 Å are nearly equivalent to the PMF with no external force applied. However, smaller values of r_{c} lead to compression of the SA_1DMA_1 cluster, resulting in a change in the resulting PMF.

During the tests, we noticed that applying the CSVR thermostat along with the external potential led to a significant increase in the translational kinetic energy, while depleting the rotational and vibrational energy, similar to the 'flying ice cube' effect.[18] Although we were unable to pinpoint the exact cause of this issue, we suspect it is related to the global working principle of the thermostat. The local Langevin thermostat does not exhibit the same problem. As a result, we recommend the Langevin thermostat for US production runs where the external potential is applied.

In the case of $(\mathrm{methanol})_2$ + $(\mathrm{methanol})_1$ dimerization, the prevention of evaporations must be balanced with 'non-squeezing' of the cluster. This requires finetuning, which is beyond the scope of the current study.

S10 Free energy convergence

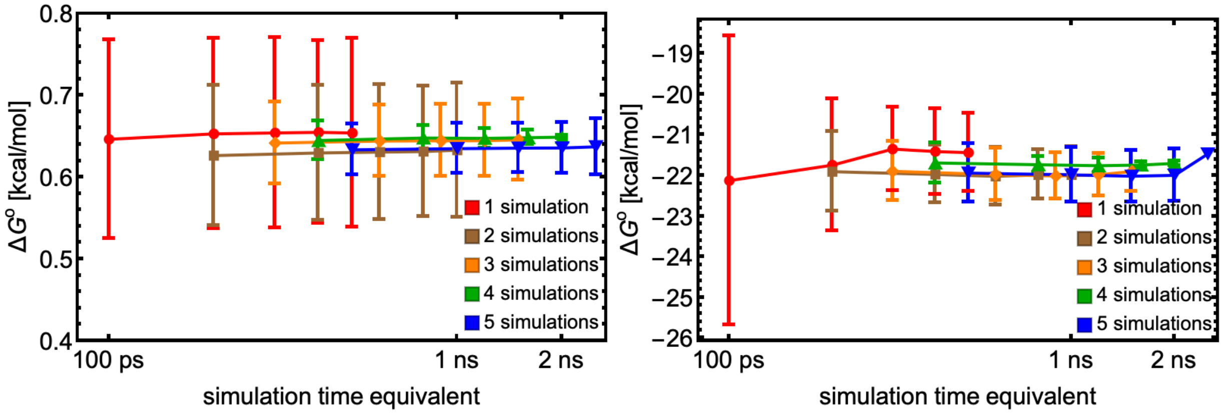

(a) $W_1 + W_1 \rightarrow W_2$ (b) $SA_1 + SA_3AM_4 \rightarrow SA_4AM_4$

Figure S10: Convergence of binding energy (ΔG) with simulation time equivalent, i.e., either running long simulations or combining multiple simulations.

To test the dependence of the calculated ΔG on the simulation duration and the number of simulations, we performed 10 independent 0.5 ns US simulations for 1) water dimerization and 2) sulfuric acid addition to the SA_3AM_4 cluster. The output from each simulation was divided into blocks of 100, 200, 300, 400, or 500 ps. For each block size, the output from 1 to 5 simulations was combined to form a single biased distribution, from which the ΔG was computed. By repeating this process for different combinations of blocks and simulations, we obtained a mean ΔG and standard deviation as a function of the simulation time equivalent, i.e., the block size multiplied by the number of included simulations.

Figure S10 shows the mean ΔG as a function of the simulation time equivalent, where error bars represent the standard deviation. For the same simulation time equivalent, the standard deviation generally decreases with the number of simulations included. While a 2 ns simulation time equivalent seems to be sufficient (errors $<$ 0.05 kcal/mol) for water dimerization, the SA_1+SA_3AM_4 system converges already at a 0.5 ns time equivalent, with no further improvements (errors of $\pm\sim$0.7 kcal/mol). To ensure well-converged ΔG and smooth PMFs, we used a combination of 10 independent 0.5 ns long simulations, resulting in a simulation time equivalent of 5 ns for the US production runs in this study.

S11 Binding free energy values

In this section, we present the numerical values of binding free energies from both experiments, US simulations, and quantum chemistry (QC) calculations.

Table S1: ΔG obtained from US simulations and traditional QC calculations at a low level of theory (XTB = GFN1-xTB) and at high level of theory (DLPNO//DFT = ωB97X-D/6-31++G(d,p) with DLPNO^{NormalPNO}-CCSD(T_0)/aug-cc-pVTZ single-point electronic energy correction). Quasi-harmonic approximation with 100 cm^{-1} threshold has been applied to all QC calculations.

Colliding system	US^{XTB}	QC^{XTB}	$QC^{DLPNO//DFT}$	US^{XTB} $-QC^{XTB}$	US^{XTB} $-QC^{XTB}$ $+QC^{DLPNO//DFT}$
2 W_1	0.91	2.31	3.22	−1.40	1.82
2 $MeOH_1$	1.37	4.37	4.11	−3.00	1.11
TMA_1+W_1	1.20	2.89	1.45	−1.69	−0.24
DMA_1+W_1	0.92	3.16	2.03	−2.24	−0.21
SA_1W_1+W_1	−2.33	−0.89	−0.27	−1.44	−1.71
SA_1+W_1	−3.35	−2.06	−1.75	−1.29	−3.04
2 FA_1	−5.48	−5.37	−2.85	−0.11	−2.96
2 ACA_1	−5.68	−5.57	−3.61	−0.11	−3.72
$B_1^-W_1$+W_1	−5.53	−2.67	−2.48	−2.86	−5.34
B_1^-+W_1	−5.70	−3.22	−3.79	−2.48	−6.27
SA_1DMA_1+W_1	−5.21	−3.22	−2.80	−1.99	−4.79
2 SA_1	−4.15	−2.83	−5.69	−1.32	−7.01
SA_1+AM_1	−12.12	−11.26	−5.86	−0.86	−6.72
SA_1+SA_3W_4	−7.61	−7.57	−5.58	−0.04	−5.62
SA_1+DMA_1	−14.77	−13.42	−11.28	−1.35	−12.63
SA_1+SA_3AM_4	−21.89	−19.62	−19.50	−2.27	−21.77
SA_1+SA_3EDA_4	−25.72	−30.21	−26.80	4.49	−22.31
B_1^-+SA_1	−28.24	−29.72	−32.68	1.48	−31.20
NT_1^-+NTA_1	−23.90	−25.49	−19.51	1.59	−17.92
SA_1+SA_3GD_4	−22.71	−40.99	−34.05	18.28	−15.77

Colliding system	US^{XTB2}	QC^{XTB2}	$QC^{DLPNO//DFT}$	US^{XTB2} $-QC^{XTB2}$	$US^{XTB2}$$-QC^{XTB2}$ $+QC^{DLPNO//DFT}$
2 W_1	1.08	2.87	3.22	−1.79	1.43
DMA_1+W_1	1.20	3.70	2.03	−2.50	−0.47
SA_1DMA_1+W_1	−5.20	−2.28	−2.80	−2.92	−5.72
B_1^-+W_1	−9.14	−6.53	−3.79	−2.61	−6.40
SA_1+SA_3AM_4	−40.03	−36.24	−19.50	−3.79	−23.29
SA_1+SA_3GD_4	−45.89	−40.15	−34.46	−5.74	−40.2

Table S2: Experimental values of Gibbs binding free energies.
Legend: W = water, DMA = dimethylamine, TMA = trimethylamine, FA = formic acid, B = bisulfate, SA = sulfuric acid

	ΔG [kcal/mol]
2 $MeOH_1$	2.02 ± 0.13,[19] 1.1 ± 0.9,[20] 1.7 ± 0.9,[21] 4.67 ± 0.06,[22] 1.7[23]
2 W_1	1.87 ± 0.21,[24] 1.78 ± 0.02,[25] 1.86 ± 0.08,[26] 2.38 ± 0.1,[26] 1.75,[27] 1.95[28]
W_1+TMA_1	1.20 ± 0.05[29]
W_1+DMA_1	0.91 ± 0.05[29]
SA_1W_1+W_1	−2.3 ± 0.3[30]
2 FA_1	−3.31 ± 0.07,[31] −3.52 ± 0.19,[32] −3.49,[33] −4.8 ± 0.8[34]
SA_1+W_1	−3.6 ± 1.0[30]
$B_1^-W_1$+W_1	−4.6 ± 1.1[35]
2 ACA_1	−5.0 ± 1.3,[36] −4.1 ± 0.9,[37] −4.48 ± 0.12[38]
B_1^-+W_1	−6.0,[39] −5.9,[40] −6.0 ± 1.0[35]

S12 PMFs of all studied systems

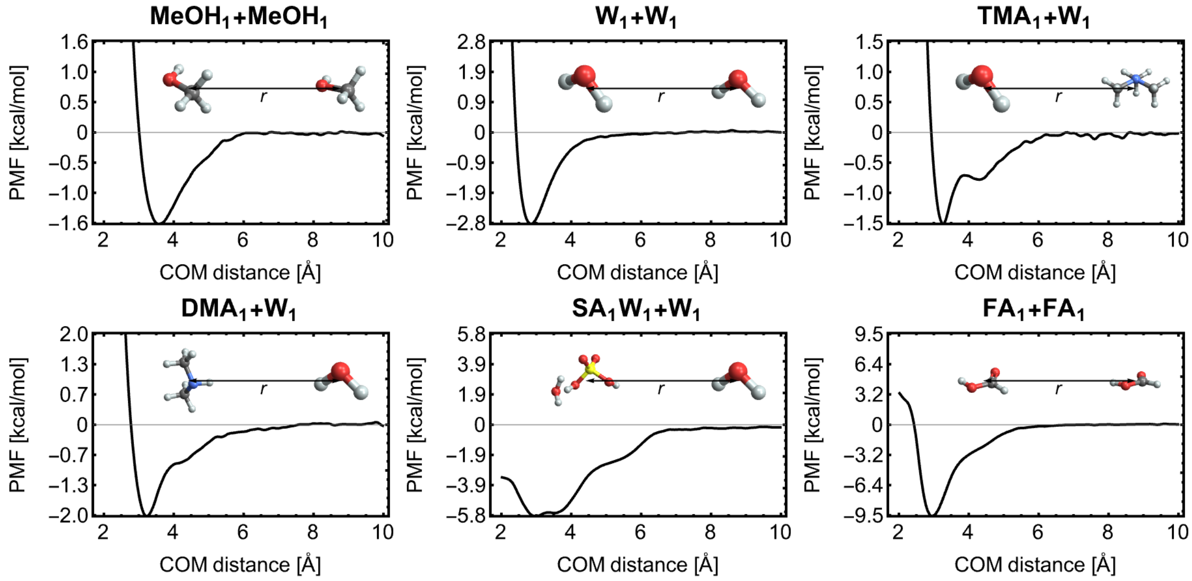

Figure S11: Potentials of mean force (PMF) obtained via umbrella sampling simulations at the GFN1-xTB level. The evaporation coordinate is the center-of-mass (COM) distance.

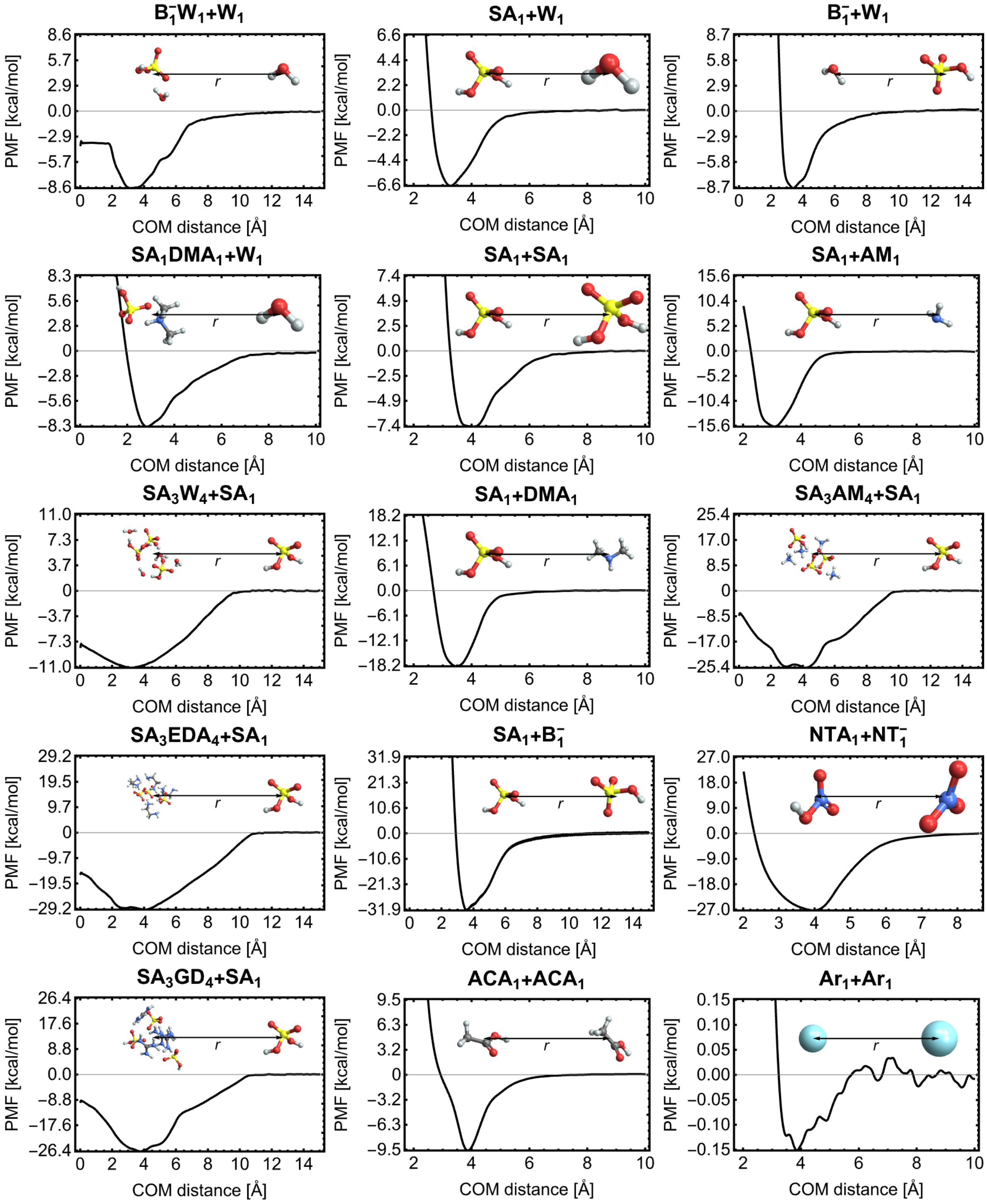

Figure S12: Potentials of mean force (PMF) obtained via umbrella sampling (US) simulations at the GFN1-xTB level. The evaporation coordinate is the center-of-mass (COM) distance.

S13 Free energy profile of W_{10}+W_1

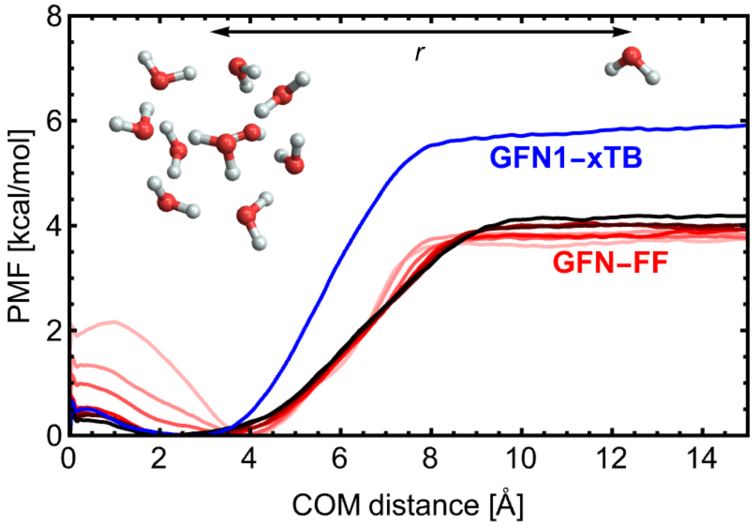

Figure S13: Potential of mean force (PMF) for W_{10}+W_1 obtained at either the GFN1-xTB or GFN-FF level. At the GFN-FF level, we used different radius cutoffs of the flat-bottom-harmonic potential = force field that prevents the cluster evaporation. The cutoff at 4.75 Å was then used for the GFN1-xTB umbrella sampling (US) simulation.

References

(1) Berendsen, H. J. C. *Simulating the Physical World: Hierarchical Modeling from Quantum Mechanics to Fluid Dynamics*; Cambridge University Press, 2007; pp 211—-248.

(2) Tuckerman, M. Advanced Statistical Mechanics: A simple example - the quantum harmonic oscillator. 2023; `https://chem.libretexts.org/@go/page/5259` [Online; accessed 2025-02-12].

(3) Kästner, J.; Thiel, W. Analysis of the statistical error in umbrella sampling simulations by umbrella integration. *J. Chem. Phys.* **2006**, *124*, 234106.

(4) Beutler, T. C.; Mark, A. E.; van Schaik, R. C.; Gerber, P. R.; van Gunsteren, W. F. Avoiding singularities and numerical instabilities in free energy calculations based on molecular simulations. *Chemical Physics Letters* **1994**, *222*, 529–539.

(5) Kästner, J. Umbrella sampling. *Wiley Interdiscip. Rev.: Comput. Mol. Sci.* **2011**, *1*, 932–942.

(6) Grest, G. S.; Kremer, K. Molecular dynamics simulation for polymers in the presence of a heat bath. *Phys. Rev. A* **1986**, *33*, 3628–3631.

(7) Bussi, G.; Donadio, D.; Parrinello, M. Canonical sampling through velocity rescaling. *J. Chem. Phys.* **2007**, *126*, 014101.

(8) Zhang, J.; Dolg, M. ABCluster: the artificial bee colony algorithm for cluster global optimization. *Phys. Chem. Chem. Phys.* **2015**, *17*, 24173–24181.

(9) Zhang, J.; Dolg, M. Global optimization of clusters of rigid molecules using the artificial bee colony algorithm. *Phys. Chem. Chem. Phys.* **2016**, *18*, 3003–3010.

(10) Halonen, R.; Neefjes, I.; Reischl, B. Further cautionary tales on thermostatting in molecular dynamics: Energy equipartitioning and non-equilibrium processes in gas-phase simulations. *J. Chem. Phys.* **2023**, *158*, 194301.

(11) Kubečka, J.; Ayoubi, D.; Tang, Z.; Knattrup, Y.; Engsvang, M.; Wu, H.; Elm, J. Accurate modeling of the potential energy surface of atmospheric molecular clusters boosted by neural networks. *Environ. Sci.: Adv.* **2024**, *3*, 1438–1451.

(12) Kingma, D.; Ba, J. Adam: A Method for Stochastic Optimization. International Conference on Learning Representations (ICLR). San Diega, CA, USA, 2015.

(13) Sparta, M.; Toffoli, D.; Christiansen, O. An adaptive density-guided approach for the generation of potential energy surfaces of polyatomic molecules. *Theor. Chem. Acc.* **2009**, *123*, 413–429.

(14) Bowman, J. M. Self-Consistent Field Energies and Wavefunctions for Coupled Oscillators. *The Journal of Chemical Physics* **1978**, *68*, 608–610.

(15) Bowman, J. M. The self-consistent-field approach to polyatomic vibrations. *Accounts of Chemical Research* **1986**, *19*, 202–208.

(16) Christiansen, O. Selected new developments in vibrational structure theory: potential construction and vibrational wave function calculations. *Phys. Chem. Chem. Phys.* **2012**, *14*, 6672–6687.

(17) Artiukhin, D. G.; Godtliebsen, I. H.; Schmitz, G.; Christiansen, O. Gaussian process regression adaptive density-guided approach: Toward calculations of potential energy surfaces for larger molecules. *The Journal of Chemical Physics* **2023**, *159*, 024102.

(18) Harvey, S. C.; Tan, R. K.-Z.; Cheatham III, T. E. The flying ice cube: Velocity rescaling in molecular dynamics leads to violation of energy equipartition. *Journal of Computational Chemistry* **1998**, *19*, 726–740.

(19) Jensen, C. V.; Vogt, E.; Poulsen, A. S.; Kjaergaard, H. G. Room temperature gas phase equilibrium constants of the methanol dimer, trimer, and tetramer. *J. Phys. Chem. A* **2024**, *128*, 6382–6391.

(20) Clague, A.; Govil, G.; Bernstein, H. Medium effects in nuclear magnetic resonance. VII. Vapor phase studies of hydrogen bonding in methanol and methanol-trimethylamine mixtures. *Can. J. Chem.* **1969**, *47*, 625.

(21) Frurip, D. J.; Curtiss, L. A.; Blander, M. Thermal conductivity measurements and molecular association in a series of alcohol vapors: Methanol, ethanol, isopropanol, and t-butanol. *Int. J. Thermophys.* **1981**, *2*, 115–132.

(22) Behera, B.; Chakraborty, S. Room temperature gas phase infrared spectra of H-bonded oligomers of methanol. *Vib. Spectrosc.* **2020**, *106*, 102981.

(23) Weltner, W. J.; Pitzer, K. S. Methyl alcohol: The entropy, heat capacity and polymer-

ization equilibria in the vapor, and potential barrier to internal rotation. *J. Am. Chem. Soc.* **1951**, *73*, 2606–2610.

(24) Leforestier, C. Water dimer equilibrium constant calculation: A quantum formulation including metastable states. *J. Chem. Phys.* **2014**, *140*, 074106.

(25) Ruscic, B. Active thermochemical tables: Water and water dimer. *J. Phys. Chem. A* **2013**, *117*, 11940–11953.

(26) Ptashnik, I. V.; Smith, K. M.; Shine, K. P.; Newnham, D. A. Laboratory measurements of water vapour continuum absorption in spectral region 5000–5600 cm^{-1}: Evidence for water dimers. *Q. J. R. Meteorol. Soc.* **2004**, *130*, 2391–2408.

(27) Scribano, Y.; Goldman, N.; Saykally, R. J.; Leforestier, C. Water dimers in the atmosphere III: Equilibrium constant from a flexible potential. *J. Phys. Chem. A* **2006**, *110*, 5411–5419.

(28) Curtiss, L. A.; Frurip, D. J.; Blander, M. Studies of molecular association in H_2O and D_2O vapors by measurement of thermal conductivity. *J. Chem. Phys.* **1979**, *71*, 2703–2711.

(29) Kjaersgaard, A.; Vogt, E.; Hansen, A. S.; Kjaergaard, H. G. Room temperature gas-phase detection and Gibbs energies of water amine bimolecular complex formation. *J. Phys. Chem. A* **2020**, *124*, 7113–7122.

(30) Hanson, D. R.; Eisele, F. Diffusion of H_2SO_4 in humidified nitrogen: Hydrated H_2SO_4. *J. Phys. Chem. A* **2000**, *104*, 1715–1719.

(31) Winkler, A.; Hess, P. Study of the energetics and dynamics of hydrogen bond formation in aliphatic carboxylic acid vapors by resonant photoacoustic spectroscopy. *J. Am. Chem. Soc.* **1994**, *116*, 9233–9240.

(32) Vander Auwera, J.; Didriche, K.; Perrin, A.; Keller, F. Absolute line intensities for formic acid and dissociation constant of the dimer. *J. Chem. Phys* **2007**, *126*, 124311.

(33) Chao, J.; Zwolinski, B. J. Ideal gas thermodynamic properties of methanoic and ethanoic acids. *J. Phys. Chem. Ref. Data* **1978**, *7*, 363–377.

(34) Lazaar, K.; Bauer, S. Conversions over low barriers. 4. Dimer/monomer dissociation in formic acid. *J. Am. Chem. Soc.* **1985**, *107*, 3769–3772.

(35) Froyd, K. D.; Lovejoy, E. R. Experimental thermodynamics of cluster ions composed of H_2SO_4 and H_2O. 2. Measurements and ab initio structures of negative ions. *J. Phys. Chem. A* **2003**, *107*, 9812–9824.

(36) Lumbroso-Bader, N.; Coupry, C.; Baron, D.; Clague, D.; Govil, G. Dimerization of carboxylic acids: A vapor phase NMR study. *J. Magn. Reson. (1969)* **1975**, *17*, 386–392.

(37) Frurip, D. J.; Curtiss, L. A.; Blander, M. Vapor phase association in acetic and trifluoroacetic acids. Thermal conductivity measurements and molecular orbital calculations. *J. Am. Chem. Soc.* **1980**, *102*, 2610–2616.

(38) Socha, O.; Dračínský, M. Dimerization of acetic acid in the gas phase-NMR experiments and quantum-chemical calculations. *Molecules* **2020**, *25*.

(39) Böhringer, H.; Fahey, D. W.; Fehsenfeld, F. C.; Ferguson, E. E. Bond energies of the molecules H_2O, SO_2, H_2O_2, and HCl to various atmospheric negative ions. *J. Chem. Phys.* **1984**, *81*, 2805–2810.

(40) Blades, A. T.; Klassen, J. S.; Kebarle, P. Free energies of hydration in the gas phase of the anions of some oxo acids of C, N, S, P, Cl, and I. *J. Am. Chem. Soc.* **1995**, *117*, 10563–10571.
